# Nanocarrier‐Based Targeting of Pattern Recognition Receptors as an Innovative Strategy for Enhancing Sepsis Therapy

**DOI:** 10.1002/adhm.202501146

**Published:** 2025-07-02

**Authors:** Eman A. Ismail, Vincent O. Nyandoro, Calvin A. Omolo, Thirumala Govender

**Affiliations:** ^1^ Discipline of Pharmaceutical Sciences College of Health Sciences University of KwaZulu‐Natal Durban Private Bag X54001 South Africa; ^2^ Department of Pharmaceutics Faculty of Pharmacy University of Gezira Wad Medani Sudan; ^3^ Department of Pharmaceutics and Pharmaceutical Chemistry School of Pharmacy Kabarak University Kabarak Private Bag 20157 Kenya; ^4^ Department of Pharmaceutics School of Pharmacy and Health Sciences United States International University‐Africa Nairobi P.O. Box 14634‐00800 Kenya

**Keywords:** NOD‐like receptors, pattern recognition receptors, sepsis, targeted nanocarriers, toll‐like receptors

## Abstract

Sepsis is a life‐threatening condition caused by an abnormal immune response to infection, leading to multiple organ failure. Despite advances in understanding its pathophysiology, effective pharmacological interventions and nanomedicines to treat sepsis remain lacking. Pattern recognition receptors (PRRs), crucial in detecting microbial toxins and triggering inflammation, are promising therapeutic targets for bacterial sepsis and related organ injuries. Nanocarriers designed to target PRRs can deliver antibiotics and anti‐inflammatory agents while modulating inflammation by inhibiting PRR‐bacterial ligand interactions. This review examines PRR‐targeted nanocarriers, focusing on Toll‐like receptors (TLRs) and NOD‐like receptors (NLRs), which recognize bacterial toxins. It evaluates the nanomaterials used, their immunomodulatory effects, and their performance in various in vitro and in vivo sepsis models. The review also discusses the strengths and limitations of current research, offering insights into optimizing nanocarriers for better therapeutic outcomes. Key challenges in translating these nanosystems into clinical practice are identified, alongside potential solutions for accelerating clinical development. In conclusion, the review highlights the potential of PRR‐targeted nanocarriers in improving sepsis treatment and emphasizes their promise for future clinical application, contingent on further refinement and optimization.

## Graphical abstract

This review highlights recent progress in nanocarriers targeting pattern recognition receptors (PRRs), including Toll‐like and NOD‐like receptors, for enhancing the treatment of bacterial sepsis and related complications. These nanomedicines deliver antibiotics and anti‐inflammatory agents while modulating immune responses. The article discusses their performance in preclinical in vitro and in vivo models and outlines key challenges and strategies for advancing toward clinics.

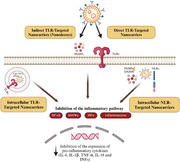

## 1 Introduction

Sepsis, a syndrome resulting from a dysregulated immune response to infection, induces damage to the body's tissues and organs, potentially leading to organ failure and death.^[^ ^1^ ^]^ According to the latest epidemiological report on the global burden of diseases, sepsis is now recognized as a significant cause of morbidity and mortality worldwide. Data from 2017 indicates 49 million cases of sepsis and 11 million sepsis‐related deaths globally, comprising ≈20% of all global deaths. The majority of cases occurred among children under five years of age, with low‐ and middle‐income countries (LMICs) reporting the highest incidences.^[^ ^2^ ^]^ Consequently, the World Health Organization (WHO) has classified sepsis as a major global health threat due to its high incidence and mortality rates, particularly in LMICs.^[^ ^3^ ^]^ These statistics underscore the urgent need for strategies aimed at improving the diagnosis and clinical treatment of sepsis.

Sepsis is characterized by two key factors: the invading pathogen and the host immune response.^[^ ^4^ ^]^ Therefore, early identification of the causative pathogen and aggressive, targeted management are essential for effective treatment. The latest International Guidelines for Management of Sepsis and Septic Shock emphasize hemodynamic stabilization, antimicrobial therapy, and, in some cases, corticosteroids as immediate and crucial measures.^[^ ^5^ ^]^ Timely administration of antibiotics has been shown to significantly improve outcomes in patients with septic shock, although this benefit may not extend to all patients with sepsis.^[^ ^6^ ^]^ This disparity may be attributed, in part, to ineffective delivery strategies of antibiotics using conventional dosage forms, which lack specificity in biodistribution and targeting of therapeutics to their site of action.^[^ ^7^ ^]^ Furthermore, conventional antisepsis regimens often fail to simultaneously control the various pathological pathways activated during sepsis, necessitating multiple therapies to manage its heterogeneous complexity. However, this approach can burden patients with more pills and side effects, leading to poor compliance.^[^ ^8^ ^]^ Hence, there is an urgent need for more innovative targeted approaches to effectively address the pathophysiological heterogeneity of sepsis.

The advent of nano‐based drug delivery systems has revolutionized the treatment of numerous diseases, including sepsis.^[^ ^9^ ^]^ Nanocarriers used for drug delivery offer significant potential due to their customizable properties, such as size, charge, surface chemistry, shape, and composition. Moreover, they can be functionalized with ligands, antibodies, and targeting molecules, enabling targeted and selective binding.^[^ ^10^ ^]^ Additionally, nano‐drug delivery systems can be tailored to enhance the biodistribution, efficacy, stability, and bioavailability of their payloads.^[^ ^11^ ^]^ These advantages have spurred research into advanced nano strategies aimed at overcoming the major limitations associated with conventional sepsis therapies.

Sepsis has a complex pathophysiology that offers multiple potential therapeutic targets.^[^ ^12^ ^]^ The sepsis cascade begins when microbial structures, such as lipopolysaccharide (LPS), lipoteichoic acid, peptidoglycan, and alpha‐hemolysin, are recognized by pattern recognition receptors (PRRs).^[^ ^13^, ^14^ ^]^ These PRRs include Toll‐like receptors (TLRs), NOD‐like receptors (NLRs), RIG‐I‐like receptors (RLRs), C‐type lectin receptors (CLRs), the receptor for advanced glycation end products (RAGE), and DNA‐sensing molecules.^[^ ^15^ ^]^ The binding of microbial ligands to PRRs activates the release of cytokines, which initiate the inflammatory response to eliminate pathogens and coordinate the adaptive immune response.^[^ ^16^ ^]^ Understanding how host immunity responds to microbial components via these PRRs is crucial for deciphering sepsis pathophysiology. Therefore, leveraging these pathways in sepsis pathophysiology could potentially facilitate the design of multifunctional targeted nanocarriers with enhanced antimicrobial, anti‐inflammatory, and antioxidant properties for combating sepsis.

Several studies have explored the use of small molecules, including polyphenols, eritoran, TAK‐242, chloroquine, and MCC950, to modulate the inflammatory response in disorders like sepsis by inhibiting PRRs.^[^ ^13^, ^17^, ^18^, ^19^ ^]^ Many of these compounds have shown significant benefits and some have advanced to clinical trials.^[^ ^13^, ^20^ ^]^ Moreover, integrating PRR modulators into nanocarriers offers added advantages, such as the co‐delivery of multiple therapeutic agents, which has shown promise in treating conditions like cancer^[^ ^21^, ^22^ ^]^, atherosclerosis,^[^ ^23^ ^]^, ischemia‐reperfusion injury^[^ ^24^ ^]^ and neuroinflammation.^[^ ^25^ ^]^ Additionally, biomimetic nanocarriers, particularly those coated with cellular membranes from macrophages, have also demonstrated potential in blocking microbial toxins from binding to PRRs.^[^ ^26^, ^27^ ^]^ These nanocarriers act as decoys by utilizing the PRRs on the membrane to capture bacterial toxins. This highlights the need for novel strategies that target both the inflammatory response and the causative pathogen in sepsis, particularly using smart, targeted nanoparticles. Currently, there is a burgeoning interest in drug delivery systems targeting PRRs, which hold promise as effective nanocarriers for treating sepsis.

Several reviews have documented the potential of various PRR modulators, particularly TLRs and NLRs, for treating various inflammatory disorders.^[^ ^19^, ^28^, ^29^, ^30^, ^31^, ^32^ ^]^ Additionally, a few reviews have explored the effectiveness of TLR‐targeted nanoparticles in cancer immunotherapy^[^ ^21^, ^22^ ^]^ and colorectal cancer^[^ ^33^ ^]^, while only one review has evaluated NLRP3‐targeted nanocarriers for inflammatory disorders.^[^ ^34^ ^]^ Despite the high morbidity and mortality rates associated with sepsis and the lack of satisfactory treatments, there is currently a gap in the literature regarding reviews on nanocarriers specifically targeting PRRs for sepsis. This review aims to address this gap and provide insights into the potential of nanocarriers targeting PRRs as a novel approach for treating sepsis.

This review provides a critical evaluation of recent advances in nano drug delivery systems aimed at targeting PRRs for the treatment of bacterial sepsis. It thoroughly explores the roles of various PRRs, particularly TLRs and NLRs, in initiating and exacerbating the inflammatory response to bacterial infections. The review categorizes nanocarriers targeting PRRs into three major groups: TLR‐targeted nanocarriers, NLR‐targeted nanoparticles, and nanoparticles acting as bacterial toxin decoys. Each category is critically assessed with a focus on nanocarrier design, characterization, and key findings. The strengths and limitations of the studies are discussed, alongside promising directions for future research. Challenges associated with these nanocarriers are addressed, and the review offers perspectives on their development and clinical application. Overall, this review provides a comprehensive analysis of the current state of nano drug delivery systems targeting PRRs for the treatment of bacterial sepsis, offering valuable insights into both current achievements and potential future directions in this promising field of research.

## 2 The Central Role of Pattern Recognition Receptors (PRRs) in the Inflammatory Cascade During Sepsis

Bacterial sepsis is the most prevalent type of sepsis. The most commonly isolated pathogens during bacterial sepsis include the gram‐positive bacteria *Streptococcus pneumoniae* and *Staphylococcus aureus* and the gram‐negative bacteria *Escherichia coli*, *Klebsiella *spp., and *Pseudomonas aeruginosa*.^[^ ^35^ ^]^ Innate immunity serves as the first line of defense to protect against invading bacteria. During infection, innate immune cells express various PRRs that detect molecules on the surface of pathogens, initiating protective immune responses and playing a role in the activation and regulation of specific immune responses.^[^ ^36^ ^]^ In this section, we will provide an overview of the major PRRs involved in the immune response against microbial invasion, the inflammatory responses associated with their activation, and therapeutic targeting strategies.

**Table** **1** outlines various PRRs involved in detecting microbial PAMPs, their cellular distribution, specific microbial ligands, and the inflammatory cascades triggered upon their activation. These PRRs are central to innate immunity and play a critical role in recognizing danger signals, including invading bacteria.^[^ ^37^, ^38^ ^]^ Components of bacterial cell walls are primary targets for PRRs. These components, expressed by invading bacteria, are collectively known as pathogen‐associated molecular patterns (PAMPs). PAMPS include LPS, peptidoglycan, lipopeptides (found in many pathogens), lipoteichoic acid (a component of the cell walls of gram‐positive bacteria), flagellin (a mobility factor in bacteria), and bacterial DNA.^[^ ^35^, ^39^ ^]^ PRRs, as shown in Table 1, are currently categorized based on their cellular localization into transmembrane receptors and intracellular receptors. The former includes TLRs and CLRs, while the latter includes NLRs, RLRs, and ALRs.^[^ ^40^, ^41^ ^]^

**Table 1:** Human pattern recognition receptors (PRRs) involved in detecting microbial PAMPs: distribution, specific ligands, and triggered cascades.

Major PRR	Sub‐class	Subcellular location and distribution	Microbial ligands	Origin of the ligand	Activated pathway	Reference
TLR	TLR1 (with TLR2)	Cellular membrane of Mo, DC, Ma, Eo, Ba	Triacyl lipopeptide soluble factors	Bacteria, *Mycobacteria*, *Neisseria meningitis*	NF‐κB, IRF, MAPKs	[42, 43, 44, 45, 46, 47]
	TLR2 (with TLR1 or TLR6)	Cellular membrane of microglia, Mo, DC, Ma, Eo, Ba, PMNs, T, B	lipoproteins, peptidoglycans, lipoteichoic acids, zymosan, mannan, porins, phenol‐soluble modulin and tGPI‐mucin	Various Gram‐positive bacteria, *Staphylococcus epidermidis*, Neisseria, Leptospira interrogans, mycobacteria		
	TLR4	Cellular membrane, endosomes and ERC of Mφ, DC, Ma, Eo	LPS	Gram‐negative bacteria		
	TLR5	Cellular membrane of IEC, Mo, DC	Flagellin	Flagellated bacteria		
	TLR6 (with TLR2)	Cellular membrane of Microglia, Mo, DC, Ma, Eo, Ba, NK	Diacyl lipopeptide, peptidoglycan. Lipoteichoic acid	Gram‐positive bacteria, Mycobacteria		
	TLR9	Endosomes of pDC, Eo, Ba	Non‐methylated CpG DNA	Bacteria		
	TLR11	Endosomes of Mφ, DC	Profilin and related proteins	Flagellated bacteria		
	TLR13	Endosomes	23s ribosomal RNA	Bacteria		
NLR	NLRC4	Cytosol of MC	Flagellin	Flagellated bacteria	NF‐κB, MAPKs, inflammasome	[48, 49, 50, 51, 52]
	NLRP1	Cytosol of EC, L, MC	Anthrax lethal toxin, Muramyl dipeptide, IpaH7.8	Mycobacteria, Bacillus anthracis, Shigella flexneri		
	NLRP3	Cytosol of Mo, Mφ, and N	Pore‐forming toxins, bacterial RNA, Nigericin, Maitotoxin, Imidaquinolone, Muramyl dipeptide,	Bacteria		
	NOD1	Cytosol of Mφ and EC	Diaminopimelate‐containing muramyl tripeptide derived from peptidoglycan	Gram‐negative bacteria		
	NOD2	Cytosol of Mo, Mφ, DC, EC	Muramyl dipeptide derived from peptidoglycan	Gram‐positive and Gram‐negative bacteria		
Others	RLRs	Cytoplasm of most cell types, as well as nucleus, and mitochondria,	RNA	RNA viruses	IRF/IFN	[53, 54]
	ALRs	Cyotosol and nucleus of all cell types	DNA	DNA viruses	Inflammasome	[54, 55]
	CLRs	Cellular membrane of Mo, Mφ, DC and N	Microbial glycans, including carbohydrates, proteins, lipids, and inorganic molecules	Bacteria, fungi and viruses	NF‐κB	[56, 57]

Mo monocyte, DC dendritic cell, Ma mastocyte, Eo eosinophils, Ba basophils, pDC plasmacytoid dendritic cell, Mφ macrophage, IEC intestinal epithelial cell, N neutrophil, L lymphocyte, PMN polymorphonuclear neutrophils, NK natural killer cell, MC myeloid cell, LPS lipopolysaccharide, NF‐κB nuclear factor κB, MAPKs mitogen‐activated protein kinases, IRF interferon regulatory factors, IFN interferon, RLRs RIG‐like receptors, ALRs Aim‐2‐like receptors (ALRs), CLRs C‐type lectin receptors.

Activation of PRRs triggers several signaling pathways that converge on key transcription factors such as nuclear factor‐kappa B (NF‐κB), activator protein 1 (AP‐1), mitogen‐activated protein kinases (MAPKs), and interferon (IFN) regulatory factors.^[^ ^40^ ^]^ The cumulative effect of PRR signaling is the differential expression of key proinflammatory cytokines and chemokines, including interleukin‐1 (IL‐1β), tumor necrosis factor‐alpha (TNF‐α), and IL‐6, as well as anti‐inflammatory genes.^[^ ^18^, ^42^, ^43^ ^]^ The resultant outcomes include changes in vascular endothelial permeability and the recruitment of immune cells to inflamed tissues, which are associated with poor prognosis.^[^ ^41^ ^]^ Such PRR‐mediated inflammatory responses must be tightly regulated: an inadequate response can increase susceptibility to infection, while an excessive response may lead to severe systemic inflammation and immune system dysfunction.^[^ ^44^ ^]^

To develop PRR‐targeted therapeutic approaches, it is essential to understand the immunological response profiles driven by the specific roles of individual PRRs. Therefore, this section provides a brief overview of the inflammatory responses associated with the activation of individual PRRs and their overactivation in sepsis in response to their respective PAMPs. Special emphasis will be placed on TLRs and NLRs, as the majority of current research focuses on these receptors. Additionally, TLRs and NLRs play key roles in recognizing bacterial PAMPs, which are central to the focus of this review.

### 2.1 Toll‐Like Receptors (TLRs)

Among the various PRRs, TLRs are the most extensively studied and play a central role in cytokine storms and sepsis. TLRs, as shown in Table 1, are categorized into two subfamilies based on their localization: cell membrane‐bound TLRs (TLR1, TLR2, TLR4, TLR5, TLR6, and TLR10) and intracellular TLRs (TLR3, TLR7, TLR8, TLR9, TLR11, TLR12, and TLR13), with TLR2 and TLR4 also present in intracellular compartments of dendritic cells (DCs), where they are essential for interleukin‐12 (IL‐12) production in response to intracellular bacteria.^[^ ^46^, ^58^ ^]^

**Figure 1:**
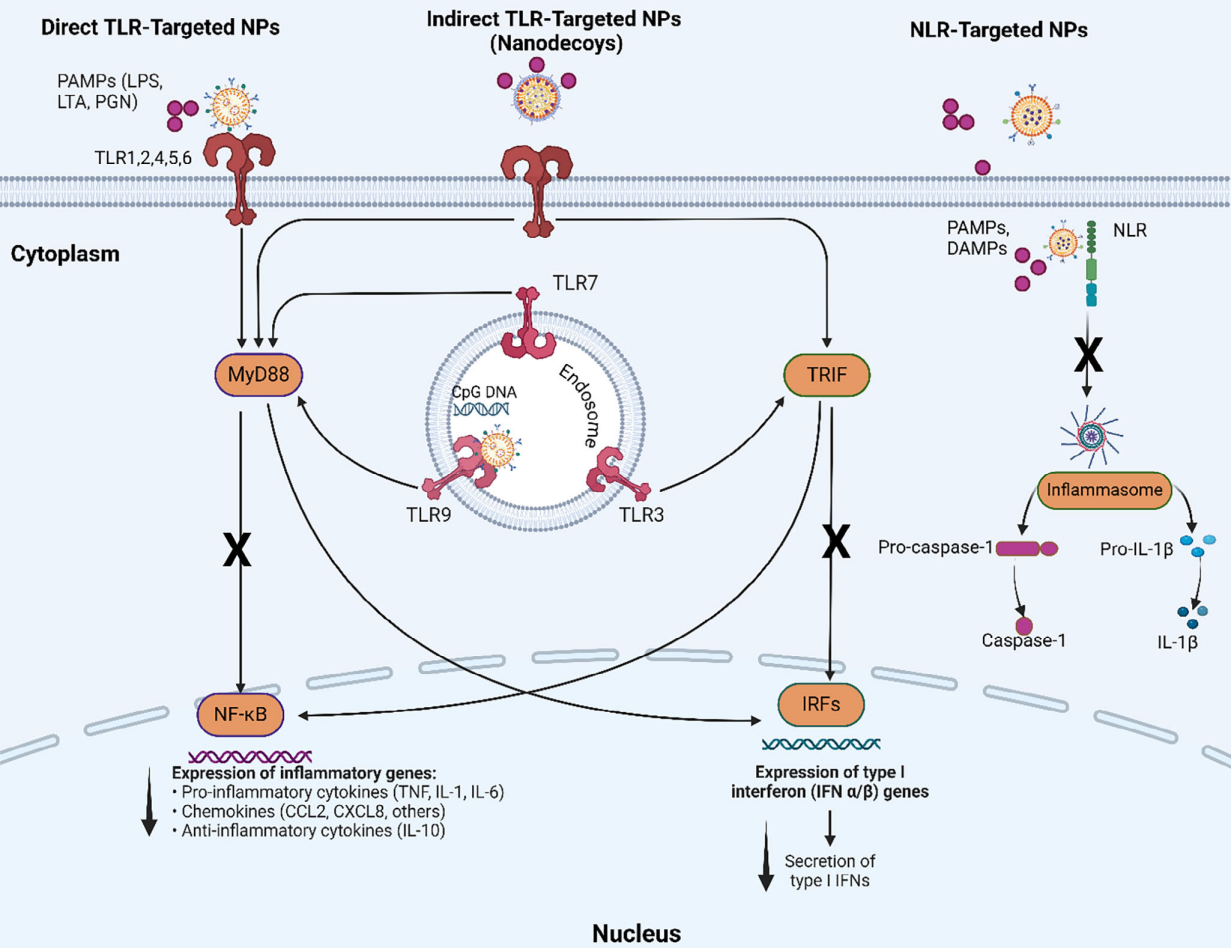
Different inflammatory pathways activated upon PAMP recognition by various PRRs, along with strategies for nanocarrier‐mediated therapeutic targeting. PAMPs, pathogen‐associated molecular patterns; LPS, lipopolysaccharides; LTA, lipoteichoic acid; PGN, peptidoglycan; DAMPs, damage‐associated molecular patterns; TLR, toll‐like receptor; NPs, nanoparticles; NLR, NOD‐like receptor; IL, interleukin; IFN, interferon; TNF, tissue necrosis factor; MyD88, Myeloid Differentiation Primary Response 88; TRIF, TIR‐domain‐containing adaptor‐inducing interferon; NF‐κB, Nuclear Factor kappa‐light‐chain; IRFs, interferon regulatory factors.

Cell surface TLRs predominantly recognize microbial membrane components, as revealed in Table 1.^[^ ^46^ ^]^ The recognition of bacterial PAMPs by their respective TLRs, as seen in **Figure** **1**, triggers the recruitment of adaptor proteins, such as Myeloid Differentiation Primary Response 88 (MyD88) and TIR‐domain‐containing adaptor‐inducing interferon‐β (TRIF), which activate downstream signaling pathways, including Nuclear Factor kappa‐light‐chain (NF‐κB), interferon regulatory factors (IRFs) or mitogen‐activated protein kinases (MAPKs) signaling pathways.^[^ ^42^ ^]^ This activation leads to the expression and release of pro‐inflammatory mediators, including cytokines, chemokines, reactive oxygen species (ROS), and reactive nitrogen species (RNS). NF‐κB activation results in the production and release of various pro‐inflammatory cytokines, such as tumor necrosis factor‐alpha (TNF‐α), interleukin‐1 alpha (IL‐1α), IL‐6, and IL‐8, which contribute to the cytokine storm and systemic inflammation during sepsis.^[^ ^43^ ^]^

Several endogenous molecules and mechanisms are known to negatively regulate TLR‐induced signaling to prevent excessive inflammation.^[^ ^46^ ^]^ Five levels of negative regulation of TLR signaling have been identified, including extracellular decoy receptors, intracellular inhibitors, membrane‐bound suppressors, degradation of TLRs, and TLR‐induced apoptosis.^[^ ^46^, ^59^ ^]^ During sepsis, however, these regulatory mechanisms are often overwhelmed, leading to dysregulated inflammation. In response to this, various studies have focused on developing small molecules to modulate and inhibit TLR hyperactivation during sepsis. For instance, TLR antagonists and signal transduction inhibitors have demonstrated significant potential in regulating the inflammatory response in various inflammatory disorders, including polymicrobial sepsis.^[^ ^13^, ^60^ ^]^

### 2.2 NOD‐Like Receptors (NLRs)

NLRs are key intracellular PRRs involved in detecting microbial infections. As demonstrated in Table 1, they are primarily expressed in the cytosol of several immune cells, as well as some non‐immune cells, including epithelial and mesothelial cells.^[^ ^49^, ^61^ ^]^ The NLR family consists of 22 molecules with a tripartite structure: a central nucleotide‐binding (NACHT) domain that mediates oligomerization, C‐terminal leucine‐rich repeats (LRRs) that bind ligands, and a variable N‐terminal domain essential for signaling. Based on their N‐terminal domains, NLRs are classified into five subfamilies: NLRA, NLRB, NLRC, NLRP, and NLRX.^[^ ^49^, ^62^, ^63^ ^]^

A variety of PAMPs detected by NLRs have been identified (Table 1). Upon detecting their specific PAMPs, NLRs activate several signaling pathways, including nuclear factor‐κB (NF‐κB) signaling, mitogen‐activated protein kinase (MAPK) signaling, and inflammasome activation.^[^ ^50^ ^]^ Similar to TLRs, activation of NF‐κB and MAPK signaling pathways leads to the activation of transcriptional programs, resulting in pro‐inflammatory responses.^[^ ^51^ ^]^ Inflammasome activation, particularly of the NLRP3 inflammasome, is one of the most studied responses following NLR activation. Inflammasomes formed by distinct NLRs, as shown in Figure 1, converge on the activation of pro‐caspase‐1 into its active form, caspase‐1. Cleaved caspase‐1 processes pro‐interleukin (IL)‐1β and pro‐IL‐18 into their mature forms, IL‐1β and IL‐18, which can then be released extracellularly, exacerbating the inflammatory response.^[^ ^64^, ^65^ ^]^ Additionally, active caspase‐1 cleaves gasdermin D, a pro‐inflammatory, lytic form of cell death that facilitates the release of mature IL‐1β and IL‐18.^[^ ^64^, ^66^ ^]^

Like TLRs, NLR activation must be tightly regulated to avoid unnecessary collateral damage once the infection is controlled.^[^ ^67^ ^]^ Various mechanisms have evolved to regulate inflammasome activation. For instance, proteins containing a single CARD domain (COPs) or a single pyrin domain (POPs), interact with the caspase‐1 CARD domain to restrict its interaction with apoptosis‐associated Speck‐like protein‐containing CARD (ASC), thereby halting inflammasome assembly.^[^ ^68^ ^]^ Similarly, INCA (inhibitory CARD) and caspase‐12 interact with caspase‐1 to prevent its recruitment to the inflammasome.^[^ ^69^ ^]^ Furthermore, anti‐apoptotic proteins such as Bcl‐2 and Bcl‐XL can bind directly to NLRP1, inhibiting its activation.^[^ ^70^ ^]^ Inspired by these natural negative feedback mechanisms, targeting NLR activation during inflammation has emerged as a potential therapeutic strategy. Inhibiting inflammasomes by targeting various NLRs involved in their activation has shown promise in improving therapeutic outcomes for several inflammation‐related disorders.^[^ ^71^, ^72^ ^]^

### 2.3 Other PRRs

Apart from TLRs and NLRs, other PRRs such as CLRs, RLRs, and ALRs play pivotal roles in the immune response against pathogens. These receptors are especially important in the development of viral and fungal sepsis.^[^ ^37^ ^]^ For example, CLRs, which are expressed on dendritic cells and macrophages, primarily recognize glycan structures on the surfaces of fungi, viruses, and bacteria. Upon recognizing these PAMPs, CLRs activate signaling pathways that lead to cytokine production, phagocytosis, and immune synapse formation, all aimed at controlling the invading pathogen.^[^ ^73^ ^]^ RLRs, including RIG‐I and MDA5, are cytoplasmic receptors that detect viral RNA. Their activation triggers the production of type I IFN, which plays a central role in the antiviral immune response by inhibiting viral replication and enhancing immune cell activation. Similarly, ALRs, which recognize cytosolic DNA from viruses, initiate the formation of the inflammasome, activating caspase‐1 and leading to the release of proinflammatory cytokines such as IL‐1β and IL‐18, thus driving the inflammatory response.^[^ ^53^, ^74^ ^]^

Given their critical role in immune defense, these PRRs must be tightly regulated to prevent excessive inflammation and tissue damage. Overactivation of these receptors is directly linked to the development of cytokine storms, a hallmark of viral and fungal sepsis. Consequently, therapeutic strategies are being developed to modulate these receptors in a controlled manner. For instance, agonists targeting CLRs are being explored as potential vaccine adjuvants, as they can enhance immune responses to viral infections.^[^ ^75^ ^]^ In contrast, modulation of RLR and ALR signaling holds promise for treating cytokine storms in viral infections and other autoimmune diseases.

### 2.4 Therapeutic Considerations

Antibiotics are essential for managing bacterial infections in sepsis, particularly during the early stages. However, no direct treatment exists for complications such as cytokine storms, dysregulated coagulation, and oxidative stress‐induced organ damage. An ideal therapeutic approach would involve a multi‐functional strategy that not only targets the pathogen but also regulates the inflammatory response and protects against organ injury.

Among different PRRs, TLRs and NLRs have garnered significant attention as potential therapeutic targets in the context of bacterial sepsis.^[^ ^15^ ^]^ While these receptors are critical for pathogen detection and immune response, their overactivation is linked to harmful outcomes, including excessive inflammation.^[^ ^36^ ^]^ Therefore, modulating the inflammatory response, either by directly inhibiting these receptors or using bacterial toxin decoys to block toxin‐receptor interactions, represents a promising and innovative approach to sepsis treatment.

Various bacterial toxin analogs have been developed as antagonists to control the dysregulated inflammation in sepsis.^[^ ^76^ ^]^ Additionally, a range of natural and synthetic compounds, such as polyphenols, are being investigated for their ability to inhibit TLR and NLR signaling pathways.^[^ ^77^, ^78^ ^]^ In the context of nano‐drug delivery systems, integrating PRR antagonists into nanocarriers loaded with antibiotics and anti‐inflammatory drugs has shown significant potential for improving sepsis treatment outcomes. This strategy enables a multifaceted treatment approach, addressing bacterial infection, oxidative stress, and inflammation in a single therapeutic platform.

Furthermore, bacterial toxin decoys offer another strategy to indirectly mitigate inflammation by preventing the binding of toxins to their PRRs. These decoys, including naturally secreted exosomes and cell membrane‐coated nanocarriers, can adsorb and neutralize bacterial toxins, thus reducing the inflammatory cascade.^[^ ^79^, ^80^ ^]^ Overall, targeting PRR pathways through nano‐drug delivery systems holds significant potential for expanding therapeutic options in sepsis, offering a more comprehensive treatment for this complex syndrome.

## 3 Nanocarriers‐Mediated PRR‐Targeting Against Sepsis and Related Organ Injury

The diverse array of PRRs on immune cells offers numerous targets for designing therapies aimed at protecting against pathogen‐induced cytokine storms in sepsis and related complications. Several PRR antagonists, particularly those targeting TLRs and NLRs, have been developed and show promise in reducing inflammatory markers in various in vitro and in vivo sepsis models.^[^ ^15^ ^]^ However, like many small‐molecule drugs, the conventional delivery methods for these inhibitors face limitations, including inadequate targeting and potential side effects.

**Figure 2:**
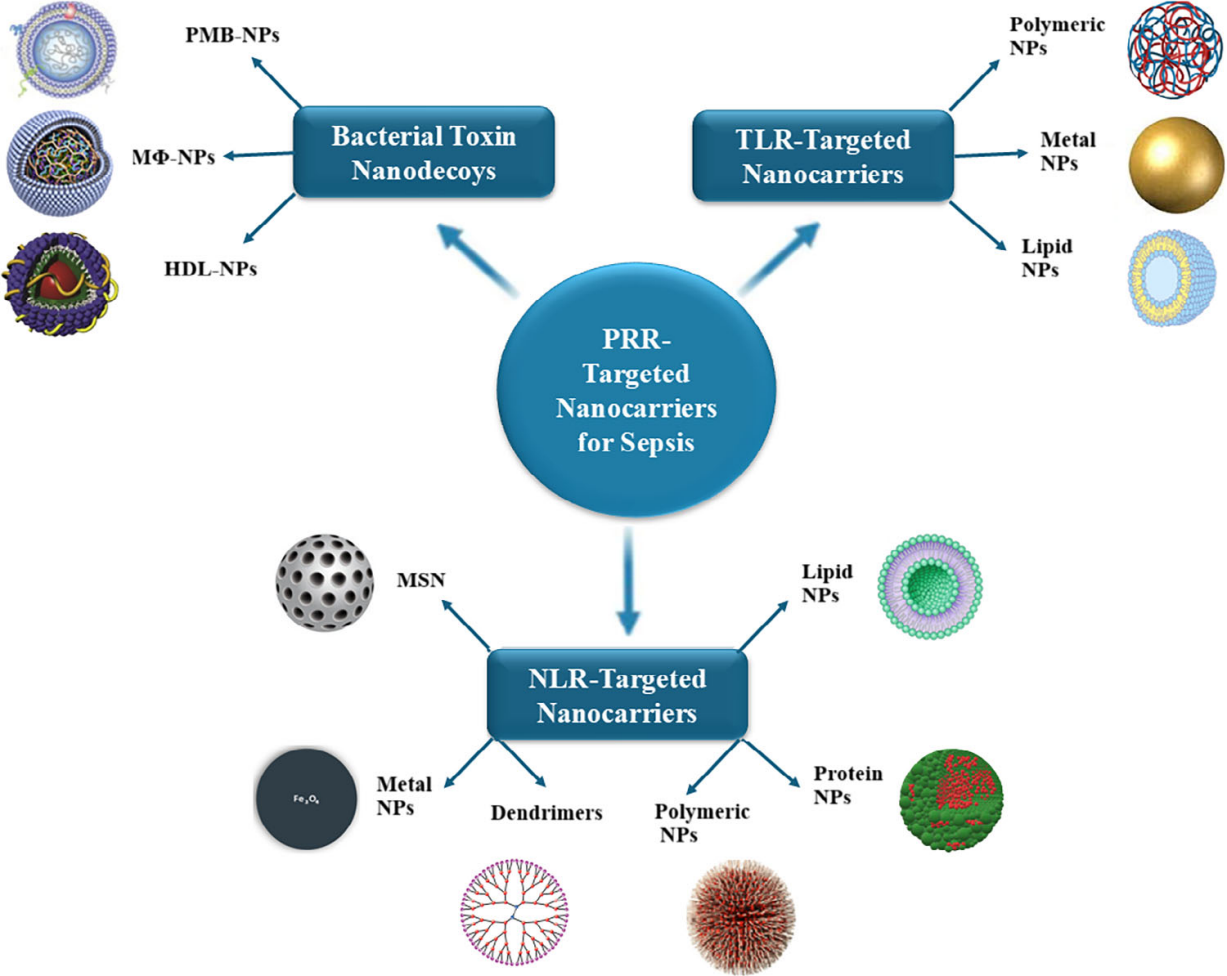
Various nano‐drug delivery systems designed to target specific PRRs to improve sepsis therapy. Some nanocarriers are cargo‐free, with inherent capabilities to bind and inhibit PRRs, while others are engineered to load PRR ligands and/or other anti‐inflammatory and antibacterial agents, ensuring targeted delivery to the sepsis microenvironment. NPs, nanoparticles; PRR, pattern recognition receptor; TLR, Toll‐like receptor; NLR, NOD‐like receptor; HDL, high‐density lipoprotein; PMB, polymyxin B; MSN, mesoporous silica nanoparticles; MΦ, macrophage.

Nanocarrier‐mediated drug delivery strategies offer a promising approach to fully utilize PRR‐targeted therapies for enhancing the treatment of sepsis and related complications. Targeted nanocarriers can accumulate preferentially in the sepsis microenvironment due to the enhanced permeability and retention (EPR) effect, as well as through ligand‐driven active targeting. Additionally, these nanocarriers can protect unstable PRR ligands from degradation and enhance their intracellular delivery by promoting endocytosis.^[^ ^81^ ^]^ In the subsequent sections, we will critically review various nanosystems designed to target PRR‐mediated inflammatory pathways involved in bacterial sepsis, with a particular focus on TLRs and NLRs. We will also explore the mechanisms through which these nanocarriers modulate the inflammatory response and examine the models used to assess their effectiveness. **Figure** **2** illustrates different nanocarriers engineered to target various PRRs to enhance the treatment of bacterial sepsis.

### 3.1 TLRs Targeted Nanocarriers

The discovery of TLRs and their role in innate immune responses has spurred considerable interest in developing drugs to control infections and improve sepsis treatment. This field is highly dynamic, with numerous TLR‐focused compounds identified and tested in both animal models and human subjects. For example, several promising TLR‐directed agents have been developed and shown considerable potential for treating autoimmune diseases and cancer.^[^ ^76^ ^]^ Moreover, various nanocarriers have been developed to modulate TLR‐inflammatory pathways, including cargo‐less nanosystems, nanocarriers encapsulating TLR antagonists, and those incorporating TLR‐targeting moieties in their design.

In this section, we focus on polymeric, metallic‐based, and lipid‐based nanocarriers that have been reported so far for targeting TLRs, with an emphasis on their design, characterization, and key findings from in vitro and in vivo models related to the modulation of TLR pathways during sepsis and sepsis‐related complications.

#### 3.1.1 Polymeric Nanocarriers

In the context of bacterial sepsis, Gram‐negative bacteria are reported as the most common causative pathogens.^[^ ^82^ ^]^ This has led to increasing interest in designing nanotherapies specifically targeting TLR4 and TLR9, responsible for recognizing LPS and CpG DNA, respectively. For instance, several studies have explored the use of polymeric nanoparticles for this purpose. **Table** **2** summarizes different polymeric nanoparticles loaded with various cargos for targeting TLR4 and TLR9, along with their characteristics and key findings regarding their potential efficacy in various sepsis models. These nanosystems have been developed both as cargo‐less nanocarriers with inherent TLR‐inhibitory activity and as carriers for targeted delivery of TLR ligands (agonists and antagonists). Notably, negatively charged polymeric nanoparticles, such as polystyrene and poly(lactic‐co‐glycolic acid) (PLGA), have been reported to preferentially bind to TLRs and be internalized by inflammatory monocytes and neutrophils following intravenous administration, offering the potential for immunomodulation in sepsis.^[^ ^83^, ^84^ ^]^

**Table 2:** Summary of the studies investigating different polymeric nanoparticles loaded with various cargo for targeting TLRs in sepsis: General aim, characteristics, tested models, type of treatment and key findings.

System	Cargo	Aim	Targeted receptor	Characterization	Tested models	Type of treatment	Main findings	Refs.
PLGA NPs PLA NPs using PEMA and PVA	–	To investigate the potential of different polymeric systems on TLR inhibition.	TLR4 TLR9	Size: 350–500 nm ZP: ‐40 to ‐50 mV (PEMA). ‐20 mV (PVA)	In vitro BMMØ, RAW 264.7 macrophages and BMDCs‐stimulated LPS. In vivo model of LPS‐induced endotoxemia, using female C57BL/6J mice.	Prophylactic and therapeutic models	○ Rapid association with BMMØ with subsequent inhibition of proinflammatory markers. ○ Significant improvement in the survival rate in septic mice.	[85]
PLA‐PVA PLA‐PEMA	–	To evaluate the mechanism of TLR‐inhibitory activity of PLA NPs	TLR4 TLR9	Size: 400– 600 nm PDI: 0.24‐ 0.28 ZP: ‐17 mV (PLA‐PVA), ‐40 mV (PLA‐PEMA)	Ex vivo model of LPS‐induced endotoxemia, using female C57BL/6J mice.	Prophylactic model	○ Decreasing activation of the NF‐κB p65 transcription factor and p38 MAPK in LPS‐stimulated BMMΦs. ○ Significant reduction of IL‐6 and TNF‐α levels.	[86]
PLGA NPs	TAK‐242	To target the delivery of a TLR4‐antagonist against sepsis‐induced AMIRI	TLR4	–	In vivo model of AMIRI using male C57BL/6J mice.	Therapeutic model	○ Enhanced circulation time. ○ Marked reduction in the infarct size, attenuation of the NF‐κB activation, and the protein levels of IL‐1β, IL‐6, and MCP‐1.	[87]
PLGA NPs	MPLA	To target the delivery of a TLR4 agonist against *E. coli*‐induced sepsis	TLR4	Size: 110 nm. EE: 49.43%	In vitro model of *E. coli*‐infected RAW 264.7. In vivo model of *E. coli*‐induced sepsis using C57BL/6 mice.	Prophylactic model	○ Improved survival rates to 60% for the 6‐h treatment, and 80% for the 24 and 48‐h treatments. ○ Reduced bacterial load across multiple organs.	[88]
PLGA NPs	ODN2088	To target the delivery of a TLR9‐antagonist against IRI‐induced AKI	TLR9	Size: 311.7 ± 12.1 nm. PDI: 0.316 ± 0.048 DL: 0.0089%	In vivo model of IRI‐AKI using male C57BL/6 mice.	Post‐exposure prophylaxis and therapeutic models	○ Reduction in the plasma blood urea nitrogen and creatinine levels. ‐Decreased kidney neutrophil gelatinase‐associated lipocalin (NGAL mRNA) expression. ○ Less tissue necrosis.	[89]
Chitosan NPs	Astragalus poly‐saccharide	To improve the bioavailability and target the delivery of a TLR4 antagonist against the CLP model of sepsis	TLR4	Size: 105 to 115 nm ZP: + 43.6 mv EE: 89.20 ± 0.77%	In vitro model of LPS‐treated H9c2 cells. In vivo model of sepsis induced by CLP in C57BL/6 mice.	Prophylactic model	○ Maintained cell viability, morphology and anti‐apoptotic effects. ○ Attenuation of sepsis‐induced MI ○ Decreased bacterial loads, CRP, and WBC levels. ○ Decreased myocardial inflammatory cytokine expression.	[90]

PLGA, poly(lactic‐co‐glycolic acid); NPs, Nanoparticles; PLA, poly(lactic acid); PEMA, poly(ethylene‐alt‐maleic acid; PVA, poly(vinyl alcohol); TLR, Toll‐like receptor; BMMØ, bone marrow macrophages; CLP, cecal ligation and puncture; ZP, zeta potential; DL, drug loading; EE, entrapment efficiency; AMIRI, myocardial ischemic reperfusion injury; IRI, ischemic reperfusion injury; AKI, acute kidney injury; MPLA, monophosphoryl lipid A; CRP, serum C‐reactive protein; WBCs, white blood cells.

**Figure 3:**
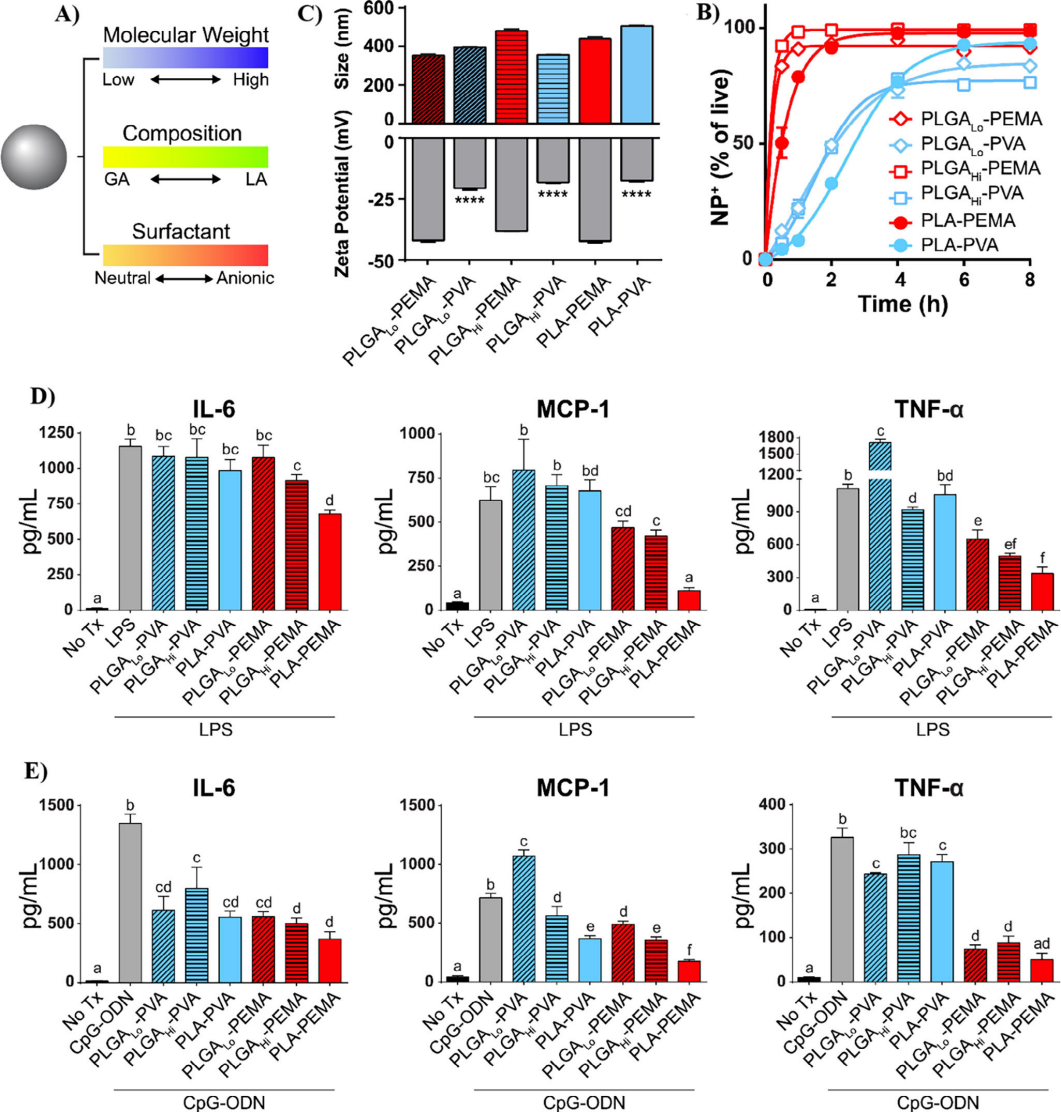
Cargo‐less nanoparticles program innate immune cell responses to toll‐like receptor activation. A) Nanoparticles were prepared utilizing polymers with different molecular weights (Low or High), compositions (glycolic acid (GA) and lactic acid (LA)), or surfactants (PVA or PEMA). B) Characterization of the prepared nanoparticles in terms of particle size and surface charge. C) kinetics of nanoparticle association with BMMØs in relation to their properties evaluated by flow cytometry. D) The effect of nanoparticles on the inflammatory cytokine secretion following stimulation by LPS, measured by ELISA. E) The effect of nanoparticles on the inflammatory cytokine secretion following stimulation by CpG‐ODN measured by ELISA. Adapted with permission.^[^ ^85^ ^]^ Copyright 2019, Elsevier.

Building on this, owing to their inherent TLR inhibitory activity, Pearson's research group conducted two studies exploring the immunomodulatory potential of cargo‐less PLGA and poly(lactic acid) (PLA) nanoparticles in targeting TLR4 and TLR9 to mitigate endotoxemia.^[^ ^85^, ^86^ ^]^ In the first study, the group investigated the effects of six nanoparticle formulations prepared using different polymers (high molecular weight PLGAHi, low molecular weight PLGALo, and PLA) and surfactants (PEMA and PVA) on TLR4 and TLR9‐mediated immune hyperactivation. These nanoparticles had similar sizes (350–500 nm) and low polydispersity but differed in surface charge. PEMA‐coated nanoparticles exhibited a charge of −40 to −50 mV, while PVA‐coated ones had a charge of −20 mV, as shown in **Figures** **3**A and 2B. As indicated in Figures 3C and 2E, PEMA‐nanoparticles showed rapid association with bone marrow macrophages (BMMΦs), with 100% cellular interaction within 1 h and significantly reduced the expression of inflammatory cytokines (IL‐6, MCP‐1, TNF‐α) following LPS and CpG‐ODN stimulation. In an in vivo endotoxemia model, both prophylactic and therapeutic treatments with PLA‐PEMA nanoparticles improved survival rates, with 62.5% survival in prophylactic and 13.5% in therapeutic treatments, suggesting their potential as protective agents in endotoxin‐induced inflammation.^[^ ^85^ ^]^ The second study focused on the molecular mechanisms of PLA nanoparticles, showing that their anti‐inflammatory activity was due to direct interaction with BMMΦs rather than sequestration of PAMPs. PLA nanoparticles reduced NF‐κB p65 and p38 MAPK activation, and modulated cytokine expression.^[^ ^86^ ^]^ Overall, both studies highlight the potential of PLGA and PLA nanoparticles as promising strategies for modulating the inflammatory response in sepsis, although further research is needed to assess their biocompatibility and pharmacokinetics. Future work should also explore combining these nanoparticles with antibiotics and anti‐inflammatory drugs to create a multi‐modal therapeutic approach, potentially improving sepsis treatment outcomes.

Further investigations have also explored the role of PLGA nanoparticles in improving the delivery of TLR4 and TLR9 ligands for treating sepsis and sepsis‐related organ injuries. For example, Fujiwara et al. utilized PLGA nanoparticles to deliver TAK‐242, an inhibitor of the TLR4 intracellular domain, to attenuate TLR4‐mediated inflammation in myocardial ischemia‐reperfusion (IR) injury.^[^ ^87^ ^]^ In a different approach, Zhao et al. used PLGA nanoparticles to deliver monophosphoryl lipid A (MPLA), a TLR4 agonist, to enhance the immune response against *E. coli*‐induced sepsis.^[^ ^88^ ^]^ While both studies demonstrated significant potential in modulating TLR4‐mediated inflammation for enhancing sepsis therapy, their approaches differed: the first focused on antagonizing TLR4 to manage inflammation, while the second aimed to enhance the TLR4‐induced response to combat sepsis. The latter approach, while potentially effective, carries the risk of exacerbating immune system paralysis and chronic inflammation. Therefore, caution is warranted when augmenting the inflammatory response in sepsis therapy. Furthermore, the second study demonstrated superiority in reducing bacterial loads in multiple organs, highlighting their potential to enhance immune cell function and strengthen the inflammatory response against the pathogen. In a more recent study, Han et al. used PLGA nanoparticles to deliver ODN2088, a TLR9 antagonist, to renal tubular cells in a model of ischemic reperfusion injury (IRI)‐induced acute kidney injury (AKI). Coating the nanoparticles with polyethylene glycol (PEG) enhanced their biocompatibility and targeting ability, resulting in a 30‐fold increase in kidney accumulation compared to free ODN2088. Treatment with ODN2088‐loaded nanoparticles significantly improved kidney function, as evidenced by reduced plasma markers (blood urea nitrogen and creatinine), decreased NGAL mRNA expression, and less tissue necrosis. Furthermore, the nanoparticles effectively reduced immune cell infiltration and proinflammatory cytokine release, offering a novel therapeutic strategy for AKI.^[^ ^89^ ^]^ Although this study used a model of IRI‐induced AKI rather than infection‐induced AKI, it converges on several key innate immune signaling pathways, including TLR9‐mediated responses, making it a promising candidate for septic‐AKI therapy. Nonetheless, further studies are needed to validate this hypothesis and confirm the efficacy of ODN2088‐loaded nanoparticles in appropriate sepsis‐induced AKI models. In summary, PLGA nanoparticles demonstrate significant potential in targeting the delivery of various TLR ligands to combat sepsis and related complications, offering promising therapeutic strategies. However, further optimization is necessary to enhance their biocompatibility, stability, and overall efficacy.

Apart from PLGA nanoparticles, Ma et al. explored the potential of polymeric chitosan nanoparticles to improve the absorption and the oral bioavailability of Astragalus polysaccharides (APS), which are known for their TLR4 inhibitory activity, against cecal ligation and puncture (CLP)‐induced sepsis and myocardial injury.^[^ ^90^ ^]^ Their results revealed that treatment with APS nanoparticles maintained the cell viability and cell morphology and exerted anti‐apoptotic effects in an in vitro model of LPS‐treated H9c2 cells. Additionally, APS‐loaded nanoparticles significantly alleviated sepsis‐induced myocardial injury in an in vivo CLP‐induced sepsis model, reducing bacterial loads, serum C‐reactive protein (CRP) and white blood cells (WBC) levels, and improving myocardial histopathology. The system remarkably reduced the myocardial inflammatory cytokine expression and inhibited the activity of the TLR4/NF‐κB pathway. The findings suggest that APS nanoparticles could protect against sepsis‐induced cardiac dysfunction by modulating the TLR4/NF‐κB pathway.

Collectively, these studies highlight the potential of polymeric nanoparticles, particularly PLGA and PLA, in enhancing sepsis treatment. These nanoplatforms can modulate immune responses by interacting with TLRs, preventing the activation of inflammatory cascades, and enabling targeted delivery of immunomodulatory agents, such as TLR antagonists. Future research is needed to optimize these nanotherapies, ensuring their biocompatibility, safety, and stability for clinical applications. Additionally, there is a notable lack of mechanistic studies investigating how nanocarrier‐mediated modulation affects inflammatory responses through TLR9. Addressing this gap is crucial for advancing the understanding of their mechanisms. Furthermore, exploring other negatively charged polymeric nanocarriers, such as poly(acrylic acid) (PAA) nanoparticles, as well as other polymeric nanocarriers, could further expand therapeutic options for modulating TLR pathways and improving the treatment of sepsis and other inflammatory disorders.

#### 3.1.2 Metallic Nanocarriers

Metallic nanoparticles, particularly gold (AuNPs) and silver (AgNPs), have emerged as promising candidates for the treatment of various biomedical conditions, including sepsis and sepsis‐induced acute lung injury (ALI). Their distinct physicochemical properties, such as high surface‐to‐volume ratio, ease of functionalization, and biocompatibility, make them ideal for targeting specific immune pathways involved in inflammation.^[^ ^91^ ^]^ This section reviews recent studies investigating the application of metallic nanoparticles, focusing primarily on gold and zinc‐based nanoparticles, in modulating TLR‐mediated inflammation in sepsis and related complications. **Table** **3** summarizes studies investigating different metallic nanoparticles employed to target TLR pathways, highlighting their efficacy in both in vitro and in vivo models of sepsis.

**Table 3:** Metallic nanocarriers investigated for inhibiting TLRs and their efficacy against in vitro and in vivo models of sepsis.

System	Targeted receptor	Aim	Tested models	Type of treatment	Key findings	Refs.
Peptide‐AuNPs	TLR4	To investigate the potential of different hybrid AuNPs to modulate the TLR4‐inflammatory pathway.	In vitro model of LPS‐stimulated THP‐1.	Prophylactic model	○ P12 reduced NF‐κB/AP‐1 and IRF3 activation. ○ Reduced levels of MCP‐1, IL‐1α, GRO‐α, and MIP‐1β.	[93]
Peptide‐AuNPs	TLR4	To investigate the pharmacokinetics and pharmacodynamics of P12.	In vivo model of LPS‐induced ALI in male C57BL/6J wild‐type mice.	Therapeutic model	○ Decreased edema, fibrin deposition, hemorrhage, and reduced inflammation and necrosis. ○ Reduced infiltration of neutrophils. ○ Highest accumulation of P12 in the lungs.	[94]
Peptide‐AuNPs	TLR4	To identify the key physicochemical properties that could further strengthen the TLR‐inhibitory activity of P12.	In vitro model of LPS‐stimulated THP‐1. In vivo model of LPS‐induced ALI in male C57BL/6J wild‐type mice.	Prophylactic model	○ The size of the core AuNPs significantly affected the TLR4 inhibition. ○ AuNPs core of 20 nm downregulated the pro‐inflammatory cytokines and prolonged the survival of mice experiencing lethal LPS challenge.	[95]
Peptide‐AuNPs	TLR4	To evaluate the potential of modifying peptide‐AuNPs with CSE, for synergizing the TLR4 inhibitory activity.	In vitro model of LPS‐stimulated THP‐1. ‐In vivo model of LPS‐induced ALI in male C57BL/6J wild‐type mice.	Prophylactic model	○ 1% of CSE markedly improved the in vitro TLR4 inhibitory activity of P12. ○ Improved in vivo anti‐inflammatory effects.	[96]
Peptide‐AuNPs	TLR4	To systematically compare the effects of the administration routes on the TLR4‐inhibitory activity, biodistribution and pulmonary cell targetability of P12.	In vitro model of LPS‐stimulated BMMØ. ‐In vivo model of LPS‐induced ALI in male C57BL/6J wild‐type mice.	Prophylactic model	○ The i.t. route was superior in reducing lung inflammation. ○ i.t. route resulted in a higher accumulation of P12 in the lungs. ○ i.t. route facilitated specific targeting of pulmonary macrophages.	[97]
AuNPs	TLR2	To investigate the effect of AuNPs size on the TLR2 inhibitory activity.	In vitro model of *Leptospira*‐activated HEK‐Blue‐hTLR2 cells	Prophylactic model	○ 20 nm AuNPs significantly reduced TLR2 activation and the expression of proinflammatory cytokines.	[98]
Tannic acid‐Zn^2^⁺ complex NPs	TLR4, TLR9, TLR3	To evaluate the synergistic efficacy of loaded antibiotics into TA‐ZN NPs against sepsis.	In vitro model of CpG Bw006 or Poly (I:C), LPS‐stimulated HEK‐Blue hTLR cells, and CpG Bw006‐activated RAW 264.7 macrophages. In vivo model of CLP‐induced sepsis in C57 mice.	Therapeutic model	○ Significant inhibition of TLR9 activation. ○ ROS scavenging, and protection against ROS‐induced cell death. ○ Improvement of the survival rate in septic mice.	[99]

TLR, Toll‐like Receptor; AuNPs, Gold nanoparticles; LPS, Lipopolysaccharide; THP‐1, human leukemia monocytic cell line; NF‐κB, Nuclear factor‐κB; AP‐1, activator protein‐1; IRF3, Interferon regulatory factor 3; MCP‐1, Monocyte chemoattractant protein‐1; IL, Interleukin; GRO‐α, growth‐regulated oncogene alpha; MIP‐1β,  Macrophage inflammatory protein‐1β; ALI, Acute lung injury; CSE, Cigarette smoke extract; BMMØ, Bone marrow macrophages; i.t., Intratracheal; CLP, Cecal ligation and puncture; ROS, Reactive oxygen species.

**Figure 4:**
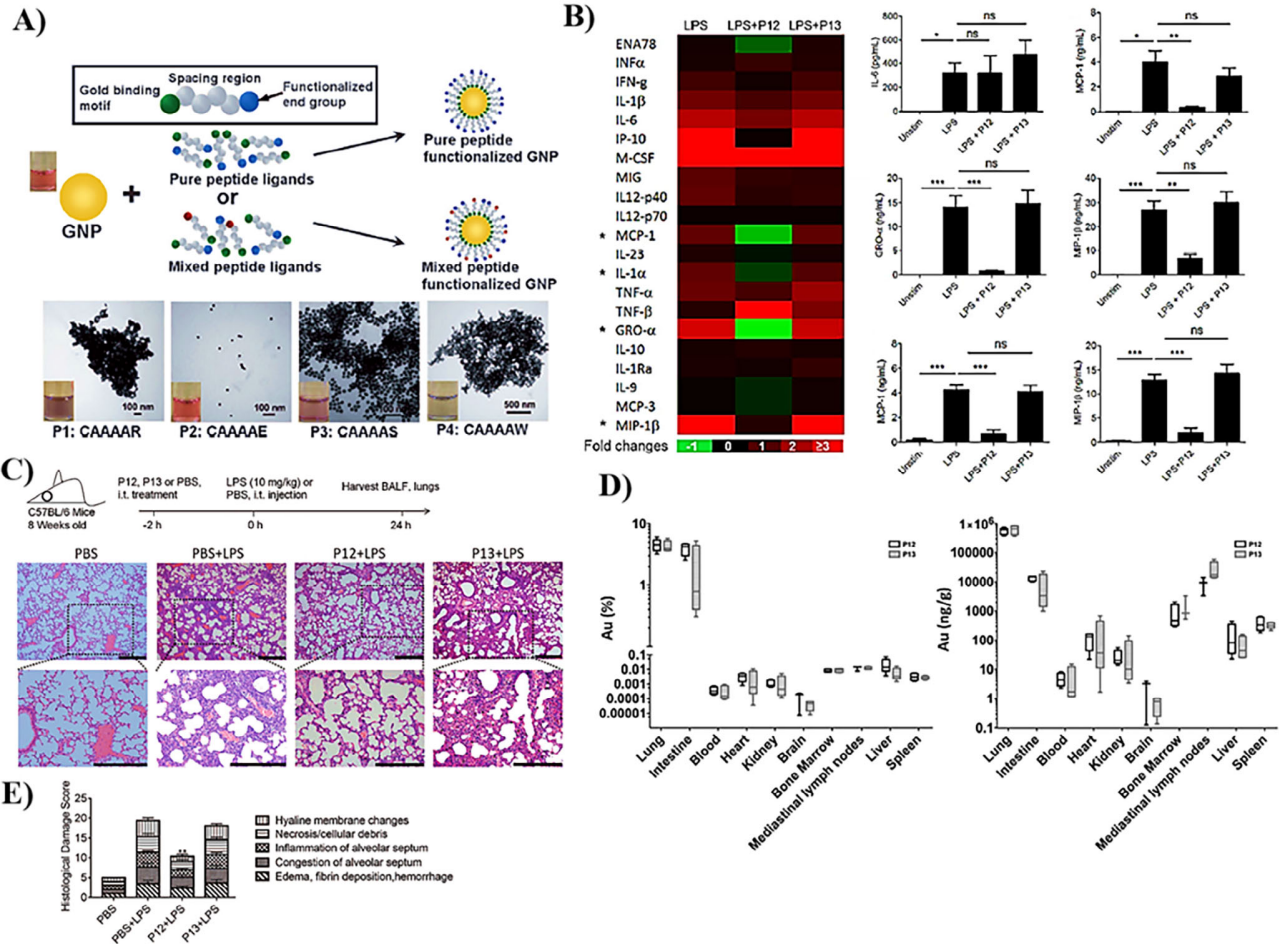
Fabrication, characterization, and evaluation of hexapeptide‐conjugated AuNPs. A) Schematic representation of the fabrication of hexapeptide‐AuNPs using single or binary peptide mixtures to achieve different surface chemical properties, followed by evaluation of their surface morphology. Adapted with permission.^[^ ^92^ ^]^ Copyright 2011, John Wiley and Sons. B) Cytokine array profile after treatment with P12, the leading potential candidate, compared to P13, the least effective candidate, in LPS‐stimulated THP‐1 cells. Adapted with permission.^[^ ^93^ ^]^ Copyright 2015, American Chemical Society. C) Protective effects of P12 on pulmonary tissue structure, as indicated by the histological damage score, compared to P13 and the negative control group. D) Biodistribution of P12 in various organs shows a high percentage of accumulation in the lungs. Adapted with permission.^[^ ^94^ ^]^ Copyright 2018, John Wiley and Sons. Values are presented as mean ± standard deviation. One‐way ANOVA with Tukey's multiple comparison test was used for statistical analysis. ns = not significant, *p < 0.05, **p < 0.01, ***p < 0.001, ****p < 0.0001.

AuNPs have been extensively studied for their ability to modulate immune responses, particularly through their interaction with TLRs. Yang et al. conducted an in‐depth investigation into the development of AuNPs for enhancing the treatment of sepsis and sepsis‐induced ALI. Their initial goal was to create physiologically stable peptide‐conjugated AuNPs with high cellular uptake. The study focused on modifying the surface properties of AuNPs by attaching various single or binary peptide ligands, as shown in **Figure** **4**A. Their results showed that AuNPs modified with a single peptide, P2, exhibited the highest stability, which was then combined with other peptide ligands to further alter the surface chemistry. The hybrid system, composed of 95% P2 and 5% P4, demonstrated superior serum stability and cellular uptake.^[^ ^92^ ^]^ In a subsequent study, Yang et al. explored the potential of these hybrid nanoparticles to inhibit TLR inflammatory signaling pathways activated by LPS. Among the ten tested peptide‐AuNP hybrids, only one, P12, significantly inhibited LPS‐induced TLR4 activation, as evidenced by reduced NF‐κB/AP‐1 and IRF3 activation. Further analysis of P12's effects on cytokine production, as seen in Figure 4B, revealed a notable decrease in MCP‐1, IL‐1α, GRO‐α, and MIP‐1β. The in vivo efficacy of P12 in LPS‐induced endotoxemia showed a significant reduction in serum IL‐6 and TNF‐α. These findings highlight the potential of P12 as a novel anti‐inflammatory nano‐therapy for sepsis treatment.^[^ ^93^ ^]^

Later in 2018, the same research group further investigated the mechanistic pharmacodynamics and pharmacokinetics of the most promising hybrid AuNPs, P12, in an in vivo model of LPS‐induced ALI. The pharmacodynamic study indicated that preventive therapy with P12 effectively alleviated LPS‐induced ALI, evidenced by decreased pulmonary injury and improved histopathology, as revealed in Figure 4C,F. On the other hand, pharmacokinetic studies, as demonstrated in Figure 4D, showed that P12 accumulated in various organs in the following order: lung > intestine >> liver > mediastinal lymph node> bone marrow > spleen ≈ heart > brain > kidney > blood, with the highest accumulation in the lungs. The substantial accumulation of P12 in the intestine suggested a potential hepatobiliary clearance route. Encouraged by these findings, the authors anticipated that this strategy would pave the way for developing next‐generation nanoimmunotherapeutics aimed at regulating both acute and chronic inflammation in the lung.^[^ ^94^ ^]^

The following year, the researchers identified the key physicochemical properties that could further strengthen the anti‐inflammatory activity of P12. These included the nanoparticle size, the density of peptide coating, as well as the number of effective amino acids in the peptide sequence. The results indicated that, among all factors evaluated, nanoparticle size significantly affected the TLR4 inhibition. Specifically, the peptide‐conjugated hybrids with AuNPs core of 20 nm (P12(G20)) exhibited the most potent inhibitory activity on TLR4 activation and its downstream pro‐inflammatory cytokines, compared to those with a core of 13 nm (P12(G13)) and 5 nm (P12(G5)). In an ALI model, P12(G20) was superior in prolonging survival, decreasing acute lung inflammation, and alleviating diffuse alveolar damage in the lungs. Detailed mechanistic studies demonstrated that when compared to the smaller P12(G13), P12(G20) had higher cellular uptake and a stronger endosomal pH buffering capacity, contributing to its enhanced anti‐inflammatory effects in vitro and in vivo.^[^ ^95^ ^]^ A similar study by Sereemaspun et al. reported the influence of nanoparticle size on the TLR2‐mediated immunomodulatory activity of AuNPs using an in vitro model of leptospirosis, a major cause of bacterial sepsis. The obtained results were consistent with the previous study, showing that 20 nm AuNPs significantly reduced TLR2 activation and proinflammatory cytokine expression.^[^ ^98^ ^]^ However, this study was limited by the lack of in vivo data to support the in vitro findings.

In a separate study, based on the hypothesis that cigarette smoke (CS) exposure might alter immune cell composition in blood and tissues, potentially providing protective anti‐inflammatory effects through TLR4 inhibition, Yang and colleagues made a serendipitous discovery. The authors hypothesized that modifying P12 with a small amount of cigarette smoke extract (CSE) could significantly enhance their TLR4 inhibitory activity. CSE‐modified AuNPs were formulated and tested for their effects on TLR4 activation in vitro and in vivo using an ALI model. The results demonstrated that CSE markedly improved the in vitro anti‐inflammatory activity of P1. In an in vivo model of LPS‐induced ALI, CSE‐P12 showed enhanced anti‐inflammatory efficacy compared to unmodified P12, as evidenced by reduced NF‐κB activation and cytokine levels.^[^ ^96^ ^]^ Overall, this study provides valuable insights into the interactions between CS components and peptide‐AuNP hybrids, along with their biological implications. It also offers new perspectives on modifying bioactive nanoparticles for potential use in treating ALI and other inflammatory diseases. However, despite these promising advantages, the use of CSE ingredients in therapeutic applications raises significant concerns. Comprehensive toxicological studies are essential, and exploring non‐toxic substitutes with similar properties should be considered for future clinical applications.

More recently, the same research group systematically compared the effects of three different administration routes, intratracheal (i.t.), intravenous (i.v.), and intraperitoneal (i.p.), on their TLR4 inhibitory activity, biodistribution, and pulmonary cell targeting features of P12. Using a mouse model of ALI, they found that the i.t. route was superior in reducing lung inflammation compared to the other two routes, even when using a lower concentration of P12. In contrast, i.v. administration led to increased nanoparticle accumulation in the liver, while i.p. administration resulted in greater accumulation in the lymph nodes, with both methods showing reduced lung accumulation. The study concluded that i.t. administration is more effective for targeting ALI with nanocarriers, as it enhances the bioavailability and efficacy of the nanodrugs in pulmonary targeted cells while minimizing potential systemic toxicity.^[^ ^97^ ^]^

Collectively, the body of research conducted by Yang and his research group provides a comprehensive evaluation of peptide‐conjugated AuNPs for their potential in treating sepsis and sepsis‐induced ALI. Through a series of innovative studies, the research team has made significant strides in enhancing the therapeutic efficacy and targeting precision of AuNPs. Future research should dive deeper into the mechanisms underlying the enhanced anti‐inflammatory activity of the nanoparticles, particularly those modified with CSE to understand the exact interactions between nanoparticles and their targets. Moreover, comprehensive toxicological evaluations are imperative to assess the long‐term safety of these nanoparticles, especially those incorporating potentially hazardous components like CSE.

In a different context, zinc (Zn^+2^)‐based metallic nanoparticles have been investigated for targeting TLR in sepsis. Leong's research group explored gentamycin‐loaded tannic acid‐Zn^2^⁺ complex nanoparticles (TA‐Zn‐Gen) to inhibit the TLR9 signaling pathway. The nanocarrier demonstrated significant in vitro anti‐inflammatory activity, including inhibition of TLR9 activation, TNF‐α transcription and translation, macrophage recruitment, ROS scavenging, and protection against cell death. In vivo results using the CLP‐induced sepsis model were consistent with the in vitro findings. The system significantly improved the survival rate to 40%, effectively inhibited TLR9 activation, and decreased TNF‐α and IL‐6 levels. Furthermore, the nanosystem significantly reduced the bacterial burden in the abdominal cavity, likely due to the targeted release of Gen, demonstrating its multifunctionality with both anti‐inflammatory and antibacterial properties.^[^ ^99^ ^]^

In conclusion, the use of metallic nanoparticles, particularly AuNPs and zinc‐based nanoparticles, represents a promising approach for the treatment of sepsis and sepsis complications such as ALI. Studies have highlighted the potential of AuNPs to modulate TLR4 signaling, with advancements in nanoparticle design, including optimization of the size, peptide composition, and modification with CSE, further enhancing their anti‐inflammatory effects. Similarly, zinc‐based nanoparticles have shown great promise in targeting TLR9 and reducing both inflammation and bacterial load, providing a multifunctional approach to sepsis treatment. However, further optimization of nanoparticle design, as well as extensive evaluation of their biodistribution, biocompatibility, and toxicity, is essential to translate these nanoparticles into clinical applications. Additionally, expanding research into other metallic nanoparticles, such as silver, copper, and iron‐based nanoparticles, presents exciting opportunities for developing new strategies to modulate TLR‐mediated inflammation in sepsis.

#### 3.1.3 Lipid‐Based Nanocarriers

Lipid‐based nanoparticles (LNPs) have gained significant attention as versatile drug delivery systems due to their ability to encapsulate a broad range of therapeutic agents, including small molecules, nucleic acids, and monoclonal antibodies.^[^ ^100^ ^]^ These nanocarriers are particularly advantageous in the context of bacterial sepsis, where they can target TLRs and modulate the immune response. This section reviews the current research on the application of LNPs for targeting TLRs against sepsis, with a focus on their role in inhibiting TLR activation and enhancing the delivery of anti‐inflammatory and antimicrobial agents.

For instance, Ferrer et al. investigated the use of liposomal spherical nucleic acids (LSNAs) for dual‐target delivery of INH‐18, a nucleic acid known to inhibit TLR9, and TAK‐242, a TLR4 antagonist. The DNA shell of INH‐18 enables enhanced cellular uptake, allowing for efficient engagement with TLR9 within endosomes. On the other hand, TAK‐242, encapsulated within the liposome, specifically targets and inhibits the intracellular domain of TLR4. The study demonstrated that LSNAs significantly enhanced the inhibitory effects on TLR9 and TLR4. In fact, LSNAs resulted in up to a 10‐fold increase in TLR9 inhibition and a 1000‐fold increase in TLR4 inhibition compared to the individual free inhibitors. Importantly, the use of TAK‐242‐loaded LSNAs also led to a marked reduction in pro‐inflammatory cytokines like IL‐6 and TNF‐α, suggesting that these nanoparticles can effectively modulate the immune response in sepsis.^[^ ^101^ ^]^ Although these results are promising, we emphasize the need for further preclinical evaluations to test the efficacy of LSNAs in different sepsis models and explore optimal treatment regimens for clinical application. In a related study, another research group explored the potential of multifunctional biomimetic liposomes incorporating a novel TLR4‐targeting peptide designed to modulate the inflammatory response while simultaneously enhancing the delivery of vancomycin against sepsis. This approach is distinguished by its dual action: anti‐inflammatory effects through the modulation of the TLR4 inflammatory pathway by the targeting peptide (P3) and antibacterial effects provided by the targeted antibiotic delivery (vancomycin). The binding affinity between P3 and TLR4 was confirmed using in silico tools and in vitro microscale thermophoresis (MST). In an in vivo model of methicillin‐resistant *Staphylococcus aureus* (MRSA)‐induced sepsis, the nanocarrier significantly reduced the MRSA burden in blood and various organs and decreased the plasma levels of inflammatory markers, including IL‐6 and TNF‐alpha and IL‐1β.^[^ ^102^ ^]^ Overall, this system represents a potentially precise approach for targeted antibiotic therapy. Future research could explore the molecular mechanism of the anti‐inflammatory activity of the systems in different models and their potential to load a broader range of broad‐spectrum antibiotics or other therapeutic agents (e.g., immunomodulatory drugs), which could further expand their clinical utility in treating sepsis.

**Figure 5:**
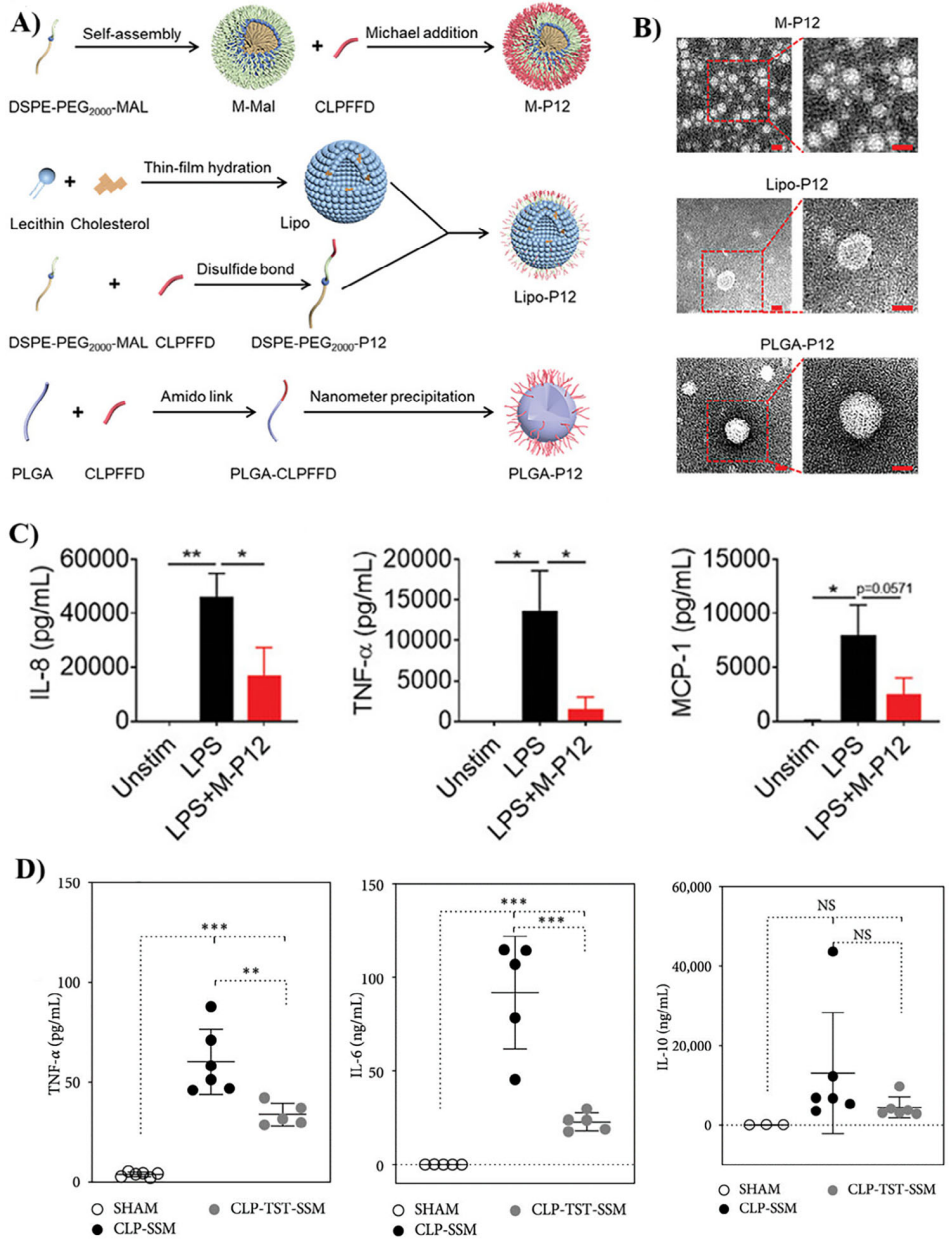
Fabrication and evaluation of TLR‐modulatory activity of a lipid‐core nanomicelle against sepsis. A) Fabrication and characterization of the hexapeptide‐modified nano‐hybrids based on biodegradable cores: M‐P12, Lipo‐P12 and PLGA‐P12 with potential inhibitory activity on TLR4 signaling. B) Morphological properties revealed via the TEM imaging of M‐P12, Lipo‐P12, and PLGA‐P12 with a zoomed‐in field on the right; scale bar = 20 nm. C) Inhibition of IL‐8, TNF‐*α*, and MCP‐1 production by M‐P12 upon LPS stimulation for 24 h in THP‐1 cell‐derived macrophages; *N*  = 3 for IL‐8 and TNF‐*α*, *N*  = 5 for MCP‐1. Adapted under the terms of the Creative Commons CC By 4.0 license.^[^ ^103^ ^]^ Copyright 2023, Yang et al. D) Cytokine profile levels in the plasma of mice with CLP‐induced sepsis quantified using ELISA kits following treatment with TST‐SSM. Adapted under the terms of the Creative Commons CC By 4.0 license.^[^ ^104^ ^]^ Copyright 2023, Esparza et al. Values were presented as mean ± standard deviation. One‐way ANOVA with Tukey's multiple comparison test was used to analyze the results. ns = not significant, *p < 0.05, **p < 0.01, ***p < 0.001, ****p < 0.0001.

In another study, Yang's research group explored the use of a biodegradable lipidic core, as an alternative to AuNPs, which are non‐biodegradable, to conjugate the TLR4 inhibitory peptide. Following an initial screening, the team identified that the peptide‐coated lipid‐core (M‐P12), constructed by the self‐assembly of distearoyl‐phosphatidylethanolaminepoly(ethylene glycol) (2000)‐maleimide (DSPE‐PEG2000‐MAL), nanomicelles (M‐P12) exhibited the most effective inhibition of TLR signaling and pro‐inflammatory cytokine production in macrophages, as shown in **Figure** **5**C. In addition, M‐P12 also significantly reduced inflammatory cells in the bronchoalveolar lavage fluid (BALF) and decreased lung injury in an LPS‐induced ALI model, indicating its effectiveness in controlling sepsis‐related acute inflammation.^[^ ^103^ ^]^ Similarly, Esparza et al. explored the potential of the same lipid core to enhance the delivery of thiostrepton (TST), a potent TLR9 inhibitor. TST is a very hydrophobic molecule, and its successful delivery is limited by its poor bioavailability and in vivo instability. To address this, the authors encapsulated TST into nanoassemblies of DSPE‐PEG2000 (TST‐SSM) to improve its pharmacokinetics and enable targeted delivery against polymicrobial sepsis (CLP). Obtained results showed that a single i.p. dose of TST‐SSM (20 mg/kg) extended the median survival time. Similar to the previous study, the treatment also reduced systemic pro‐inflammatory markers IL‐6 and TNF‐α (Figure 5D). Additionally, there was a significant reduction in the recovered bacterial load in blood and peritoneal lavage, suggesting that, in addition to its immunomodulatory effects, TST‐SSM preserves TST's antimicrobial properties.^[^ ^104^ ^]^

In conclusion, LNPs offer a promising platform for modulating the inflammatory response in sepsis, thanks to their inherent TLR‐modulatory efficacy. These nanocarriers can effectively enhance and target the delivery of various hydrophobic anti‐inflammatory agents and antibiotics for sepsis treatment. To date, research has primarily focused on liposomes and lipid‐core nanomicelles for their TLR‐modulatory effects. Future studies should explore the potential of other LNPs, such as solid lipid nanoparticles (SLNs) and nanostructured lipid carriers (NLCs). Incorporating specific lipids, such as the omega‐3 fatty acids eicosapentaenoic acid (EPA) and docosahexaenoic acid (DHA), known for their TLR‐modulatory properties, could further enhance the therapeutic potential of these nanocarriers. Additionally, optimizing production methods for scalability and conducting comprehensive evaluations of the pharmacodynamics and pharmacokinetics of these nanosystems will be critical for advancing their clinical development.

### 3.2 Indirect TLRs Targeted Nanocarriers (nanodecoys)

In addition to directly interacting with and inhibiting TLRs, nanocarriers can be engineered to indirectly target TLR pathways by scavenging the natural ligands of these receptors. This mechanism prevents the recognition of bacterial toxins by PRRs, thereby mitigating the inflammatory response. Biomimetic nanoparticles, which draw inspiration from natural biological systems to achieve desired functional properties, have been widely employed for this purpose.^[^ ^105^ ^]^

This promising strategy involves a top‐down fabrication approach where natural cellular membranes are coated onto synthetic nanocarriers.^[^ ^106^ ^]^ This technique offers several advantages: i) active targeting through interactions with membrane proteins, ii) enhanced biocompatibility and self‐recognition, which aids in evading clearance by the phagocytic system, thereby extending the circulation time, and iii) toxin decoy properties due to the presence of various PRRs on the membrane that capture bacterial toxins (PAMPs).^[^ ^107^, ^108^ ^]^ In this setup, the nanocarrier core provides stability, while the cell membrane coating confers the functional properties of the original cells.^[^ ^109^ ^]^

In the context of sepsis, cell membranes from red blood cells (RBCs), leukocytes, and platelets have been used to coat nanoparticles in order to inhibit TLR‐mediated inflammatory pathways by scavenging PAMPs.^[^ ^107^ ^]^ These membranes express a variety of receptors, including TLRs, which are crucial for their toxin‐scavenging activity. This section discusses the design, characterization, and potential therapeutic effects of nanocarriers that act as bacterial toxin decoys in sepsis and sepsis‐related complications. The studies reviewed, summarized in **Table** **4**, are categorized by the materials used as bacterial toxin decoy, including macrophage cell membranes, apolipoprotein A‐I, and polymyxin B, with a focus on their design, mechanism of bacterial toxin scavenging and efficacy in various in vitro and in vivo sepsis models.

**Table 4:** Summary of bacterial toxin nanodecoys indirectly targeting TLRs, tested models and major findings.

System	Toxin decoy	Core	In vitro cell‐tested models	In vivo models	Inoculum dose	Type of treatment	Major findings	Refs.
MΦ‐PLGA NPs	MΦ	Polymeric PLGA NPs	LPS‐stimulated HEK‐Blue mTLR4 cell	LPS‐induced endotoxemia in male BALB/c mice, *E. coli*‐induced bacteremia model in C57BL/6	5 µg/kg (LPS), 1 × 10^7^ CFU (*E. coli*)	Therapeutic model	○ Potent in vitro and in vivo neutralization of LPS. ○ Reduction in the major proinflammatory cytokine levels. ○ Significant enhancement in the survival rate.	[110]
PEG‐Mac@NPs	MΦ	Polymeric PEG NPs	LPS‐challenged peritoneal macrophage	LPS‐induced endotoxemia in female BALB/c mice.	1 mg/kg	Post‐exposure prophylaxis model	○ The system remarkably reduced the release of nitric oxide, TNF‐α, IL‐6, iNOS, and COX‐2.	[111]
Fe3O4@MMs	MΦ	Metallic Fe3O4 NPs	LPS‐activated RAW264.7 macrophage cells.	LPS‐induced endotoxemia in male ICR mice.	15 mg/kg	Post‐exposure prophylaxis model	○ Effective in vitro LPS scavenging activity. ○ Sequestration of TNF‐α and IL‐6. ○ Protection against oxidative stress‐induced injury. ○ Marked improvement in the overall survival.	[112]
MCeC@MΦ	MΦ	Metallic MSN‐ CeO2 NPs	LPS‐treated J774 macrophages	lethal doses of MDR E. coli‐induced sepsis in female BALB/c mice.	2 × 10^8^ CFU/mouse	Therapeutic model	○ Potent in vitro endotoxin neutralization and proinflammatory cytokines sequestration. ○ Effective ROS scavenging activity. ○ High antibacterial and antibiofilm efficacy. ○ Reduction in the major proinflammatory cytokine levels. ○ Significant enhancement in the overall survival rate in vivo.	[113]
M_AMPNP	MΦ	AMP‐grafted self‐assembled polymeric NPs	N/A	CLP model of polymicrobial sepsis in male C57BL/6 mice	N/A	Therapeutic model	○ Enhanced antibacterial and antibiofilm activity against Gram‐positive and negative bacteria. ○ Reduction in the levels of TNF‐α, IL‐1β and IL‐6 in CLP model. ○ Effective reduction of sepsis‐induced multiple organ damage. ○ Improved survival rates.	[26]
HDL‐like NPs	Apo A‐I	Citrate‐stabilized AuNPs	‐LPS‐stimulated THP1‐XBlue™‐MD2‐CD14. ‐Bacterially‐infected THP1‐XBlue™‐MD2‐CD14	–	–	Post‐exposure prophylaxis model	○ Significant suppression of the NF‐κB signaling following LPS stimulation. ○ Suppression of the LPS and live bacteria‐induced expression of pro‐inflammatory cytokines and chemoattractants. ○ Efficient binding to LPS.	[114]
PMB (D‐TZP) NPs	PMB	self‐assembled tannic acid (TA)/Fe3 complex	‐LPS‐activated THP1‐XBlue‐MD2‐CD14.	LPS‐induced endotoxemia in C57BL/6 mice.	20mg/kg	Prophylactic and therapeutic models	○ Significantly reduced the toxicity of PMB in vitro and in vivo. ○ Potent in vitro antibacterial activity. ○ Reduction in the level of IL‐10 and TNF‐α. ○ Improvement in overall survival.	[115]

MΦ, Macrophage; PLGA, poly(lactic‐co‐glycolic acid); NPs, Nanoparticles; LPS, Lipopolysaccharide; PEG, Polyethylene glycol; TLR, Toll‐like receptor; TNF‐α, Tissue necrosis factor alpha; IL‐6, interleukin‐6; iNOS, Nitric oxide synthase‐2; COX‐2, cyclooxygenase‐2, IL‐10, Interleukin‐10; apo A‐I, apolipoprotein A‐I; PMB, Polymyxin B; HDL, High‐density lipoprotein; AMP, Antimicrobial peptide; AuNPs, Gold nanoparticles; CLP, Cecal ligation puncture; NF‐ kB, Nuclear factor kappa; N/A, not available.

**Figure 6:**
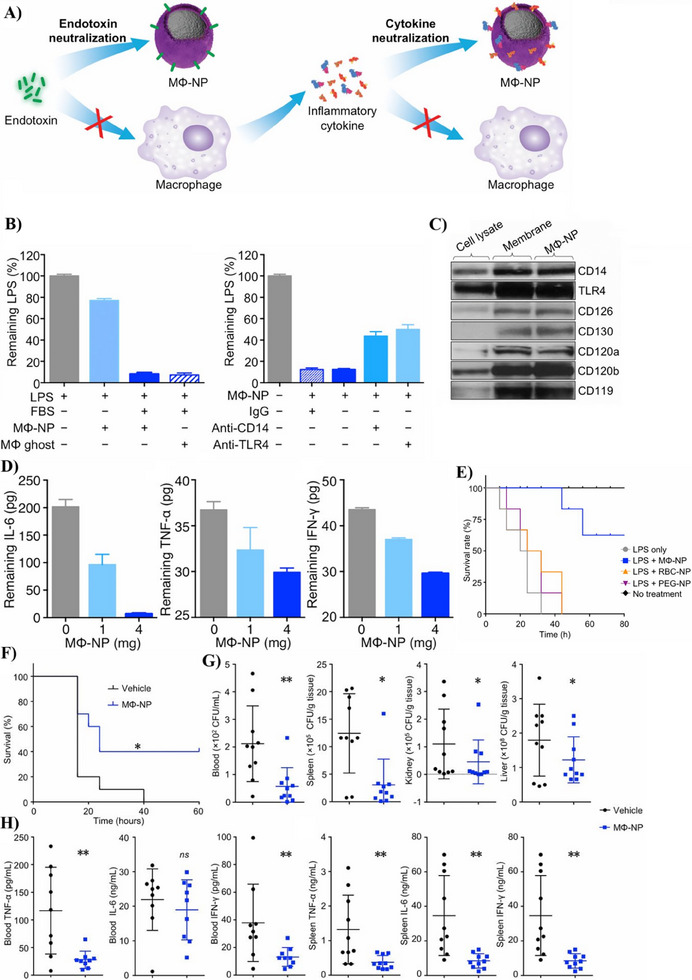
Fabrication of MΦ‐NPs as endotoxin decoys and their potential to alleviate inflammatory responses in sepsis. A) Schematic illustration of the mechanism by which MΦ‐NPs neutralize bacterial endotoxin. B) Neutralization capacity of MΦ‐NPs for removing LPS, with and without LPS binding protein and receptors. C) Qualitative Western blot analysis of MΦ membrane proteins. D) In vitro sequestration of pro‐inflammatory cytokines by MΦ‐NPs. E) Survival rate comparison in endotoxemic mice treated with various coated nanoparticles versus MΦ‐NPs. F) Evaluation of the therapeutic efficacy of MΦ‐NPs by measuring survival rates in mice. G) Recovered bacterial CFU/ml in blood, spleen, kidneys, and liver following MΦ‐NP treatment. H) Assessment of key inflammatory markers in an in vivo mouse model of endotoxemia post‐MΦ‐NP treatment. Values are presented as mean ± standard deviation. Statistical analysis was performed using one‐way ANOVA with multiple comparisons. ns = not significant; *p < 0.05; **p < 0.0. Adapted with permission.^[^ ^110^ ^]^

Macrophages (MΦ) serve as a primary line of defense against bacterial infections and possess a broad array of PRRs.^[^ ^105^, ^116^ ^]^ Consequently, MΦ‐coated nanocarriers have been extensively investigated for their potential to enhance the treatment of bacterial sepsis. For example, Thamphiwatana et al. were the first to introduce biomimetic nanoparticles with a biodegradable PLGA nanoparticle core coated with a macrophage membrane targeting sepsis. This system, as shown in **Figure** **6**A, capitalizes on the antigenic surface of the macrophage membrane to bind endotoxins, acting as a decoy to prevent their recognition by TLRs and blocking the inflammatory response. In vitro studies confirmed that MΦ‐coated PLGA nanoparticles could bind and neutralize LPS in a concentration‐dependent manner (Figure 6B,C). This was further supported by the system's ability to reduce the release of pro‐inflammatory cytokines, including TNF‐α and IL‐6 (Figure 6D), leading to a 60% improvement in survival in an in vivo mouse model of LPS‐induced endotoxemia and *E. coli*‐induced sepsis, as demonstrated in Figure 6E.^[^ ^110^ ^]^ Similarly, Wu et al. developed a MΦ‐coated nanocarrier using a recyclable polymeric PEG core (PEG‐Mac@NPs) to combat sepsis. Following the same concept introduced by Thamphiwatana et al., they showed that PEG‐Mac@NPs could scavenge bacterial LPS, attenuating the LPS/TLR4 inflammatory response. Their results align with the findings of the previous study and provide further evidence for the potential of MΦ‐coated nanocarriers as effective nanodecoys for sepsis therapy.^[^ ^111^ ^]^

**Figure 7:**
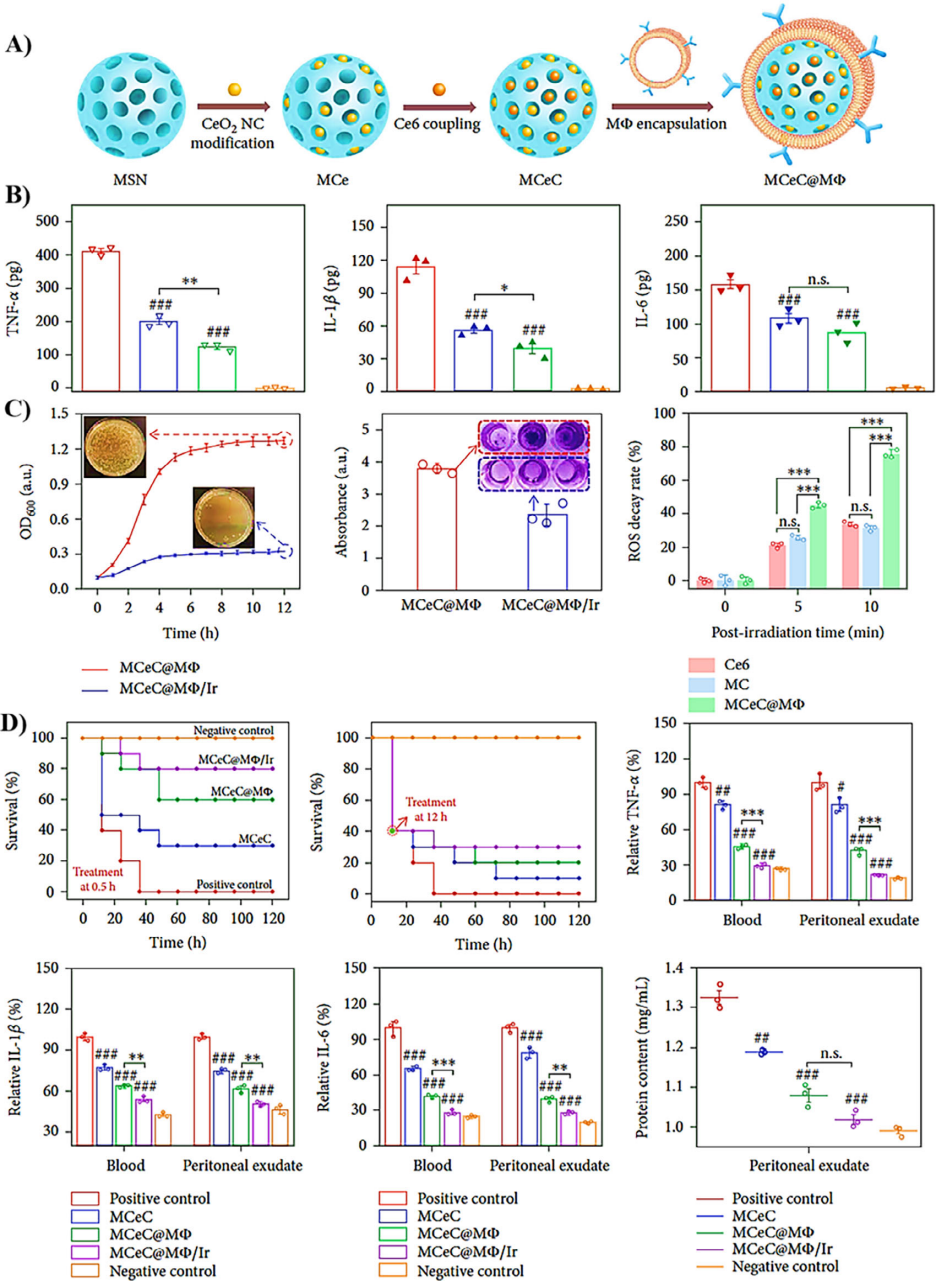
Decoy nanozyme‐enabled targeted treatment of MDR bacterial sepsis. A) Schematic illustration of the preparation method for MCeC@MΦ decoy nanozymes. B) In vitro efficacy of MCeC@MΦ decoy nanozymes for LPS neutralization, indicated by significantly reduced levels of pro‐inflammatory cytokines (TNF‐α, IL‐1β, and IL‐6) in J774 macrophage cells following treatment. C) In vitro antibacterial activity, antibiofilm properties, and ROS decay rate of MCeC@MΦ. D) In vivo performance of the nanodecoy against MDR *E. coli*‐induced sepsis in mice, showing survival rates and levels of pro‐inflammatory cytokines (TNF‐α, IL‐1β, and IL‐6) as well as protein content in the blood and peritoneal exudate post‐treatment with MCeC@MΦ. Values are presented as means from three independent experiments, with error bars indicating standard deviation. The symbol # denotes contrasts between experimental groups and the positive control. Significance levels are indicated as follows: #P < 0.05, ##/∗∗P < 0.01, ###/∗∗∗P < 0.001, and n.s. P > 0.05. Adapted under the terms of the Creative Commons CC By 4.0 license.^[^ ^113^ ^]^ Copyright 2022, Du et al.

Based on a similar mechanism, other studies have also demonstrated the potential of MΦ‐coated nanoparticles, utilizing various core particles, against various bacterial sepsis models, as detailed in Table 4. For example, one study investigated iron oxide nanoclusters wrapped in MΦ membranes for their ability to neutralize LPS and block the inflammatory cascade. The positively charged iron oxide core stabilized the macrophage membrane shell, enhanced LPS binding, and enabled magnetic separation of LPS. This system effectively absorbed LPS and reduced pro‐inflammatory cytokine production in both in vitro macrophage models and an in vivo mouse model of lethal endotoxemia.^[^ ^112^ ^]^ In another study, Du et al. used a multifunctional MΦ‐coated nanocarrier (MCeC@MΦ), incorporating mesoporous silica nanoparticles (MSNs), cerium oxide nanocatalysts (CeO2 NC), and the photosensitizer chlorin e6 (Ce6), as shown in **Figure** **7**A. This system demonstrated LPS neutralization, inhibition of pro‐inflammatory cytokines, and photodynamic eradication of multi‐drug resistant (MDR) bacteria (Figure 7B,C). The therapeutic effects in a mouse model of MDR *E. coli*‐induced sepsis showed an 80% survival rate with significant reductions in pro‐inflammatory cytokines (TNF‐α, IL‐6, IL‐1β), as demonstrated in Figure 7D.^[^ ^113^ ^]^

In addition, another group explored the use of antimicrobial peptide (AMP)‐grafted polymeric nanoparticles coated with MΦ membranes to combat bacterial sepsis. The core nanoparticle, consisting of a hydrophobic polycaprolactone (PCL) and hydrophilic poly(ethylene glycol) methyl ether methacrylate (POEGMA) block copolymer, exhibited inherent antibacterial activity due to the grafted AMP. The outer membrane shell facilitated the absorption of bacterial toxins, including LPS and lipoteichoic acid (LTA), by interacting with TLR4 and TLR2 receptors. In vitro, the system exhibited antibacterial activity against *S. aureus*, *E. coli*, and MRSA, as well as antibiofilm efficacy. In a CLP mouse model of sepsis, the system significantly reduced serum levels of inflammatory cytokines (TNF‐α, IL‐1β, and IL‐6), decreased mortality rates and protected against tissue damage.^[^ ^26^ ^]^

Collectively, these studies highlight the potential of MΦ‐coated nanoparticles as bacterial toxin decoys that indirectly block TLR‐mediated inflammatory responses. However, several challenges remain in translating these strategies to clinical practice. First, the extraction of cell membranes is complex, and the purity of the extracted membranes can vary. Second, the stability of these biomimetic nanocarriers, both in vitro and in vivo, needs further optimization. Third, the cost‐effectiveness of such systems is a significant concern. An important consideration for toxin scavengers, including nanodecoys, is determining optimal concentration and conducting thorough toxicity evaluations. These factors are crucial for the future development of nanodecoy‐based therapies. Future studies should focus on identifying the optimal concentration ranges for nanodecoys to ensure effective toxin neutralization while minimizing potential toxicity during sepsis. Additionally, research could explore extracting specific proteins and receptors involved in bacterial toxin recognition and neutralization, which could be more efficiently attached to nanocarriers, thereby enhancing the stability and effectiveness of the toxin decoys.

In addition to MΦ‐coated nanoparticles, other compounds and proteins known for their endotoxin‐neutralizing properties, such as high‐density lipoproteins (HDLs), have also been explored as bacterial toxin decoys. For instance, Foit and Thaxton engineered HDL‐like nanoparticles by modifying citrate‐stabilized AuNPs with a lipid bilayer and apolipoprotein A‐I (apo A‐I), the primary protein component of HDL. These nanoparticles effectively reduced LPS‐induced cytokine production in an in vitro model of LPS‐stimulated, and *E. coli*, *Klebsiella pneumonia*, and *Salmonella enterica*‐infected human TLR4‐reporter cell lines. The enhanced efficacy was attributed to their ability to scavenge LPS and prevent TLR4 signaling.^[^ ^114^ ^]^ Despite these promising in vitro results, the study is limited by a lack of data supporting the potential efficacy of this nanosystem in a suitable in vivo model of sepsis.

Similarly, polymyxin B (PMB), a cationic antimicrobial peptide with strong LPS‐binding and TLR4‐inhibitory properties, has been used as an endotoxin decoy. However, its clinical application is limited by nephrotoxicity and neurotoxicity. To mitigate these side effects, Yeo's group developed self‐assembled nanoplatforms incorporating PMB, tannic acid (TA)/Fe^3^⁺ complexes, and vitamin D. This system also included a succinylated chitosan derivative to reduce undesirable interactions between PMB and host cell membranes, thus attenuating its toxicity. This system effectively inactivated LPS in vitro and demonstrated improved in vivo survival rates (70% in LPS‐induced endotoxemia and 75% in CLP‐induced polymicrobial sepsis) compared to free PMB, which exhibited 100% and 67% mortality, respectively. Furthermore, the nanocarrier significantly suppressed the production of TNF‐α and IL‐10 compared to controls in the CLP model.^[^ ^115^ ^]^ This approach demonstrates the potential for enhancing the systemic tolerability of PMB while preserving its endotoxin‐scavenging and TLR4 inhibitory activities.

Overall, the indirect inhibition of the endotoxin/TLR‐mediated inflammatory response through nanodecoys represents an innovative and precise approach to improving sepsis treatment. The exploration of novel materials with high selectivity and specificity for bacterial endotoxins, coupled with enhanced in vivo biocompatibility, such as proteins derived from immune cell membranes and other small molecules like lactoferrin and benzylamine derivatives, could significantly broaden the range of available bacterial toxin traits. This expansion would facilitate the design of various nanocarriers with the potential for scaling up and translation into clinical applications.

### 3.3 NLRs Targeted nanocarriers

Various NLRs, such as NOD1, NOD2, NLRC4, AIM2, and others, contribute to the innate immune response during bacterial sepsis. However, increasing evidence has identified NLRP3 as a central mediator of excessive inflammation and pyroptosis in the early stages of sepsis.^[^ ^117^ ^]^ NLRP3 is uniquely activated by a wide range of stimuli, including DAMPs, bacterial toxins, and ATP, making it a central convergence point in the inflammatory cascade.^[^ ^118^ ^]^ Therefore, the majority of the studies reported so far focused on NLRP3, among various NLRs, as a therapeutic target due to its central role in early inflammation. Additionally, NLRP3 expression has been found to be elevated in septic patients, correlating with disease severity and adverse outcomes.^[^ ^119^ ^]^ Thus, based on its biological relevance and literature trend, in this section, we focus on NLRP3‐specific nanocarrier‐based therapy.

The activation of the NLRP3 inflammasome, a key component of this targeting approach, has been extensively studied due to its role in initiating the release of pro‐inflammatory cytokines that contribute to the inflammation seen in bacterial sepsis. Thus, targeting the NLRP3 inflammasome represents a promising strategy for modulating inflammatory responses through innovative nanosystems. Numerous studies have explored the use of various nanocarriers loaded with small molecules designed to modulate pathways associated with NLRP3 inflammasome activation. These nanosystems have demonstrated considerable promise in suppressing the expression of key components involved in NLRP3 activation, including NLRP3 itself, caspase‐1, and IL‐1β.

This section reviews the design, characterization, and therapeutic potential of different types of nanocarriers, including inorganic, protein‐based, dendrimer‐based, lipid‐based, and polymer‐based carriers, in the context of NLRP3 inflammasome inhibition against sepsis. **Table** **5** summarizes these studies, categorizing them based on the material class used, the therapeutic agents loaded, the in vitro and in vivo models employed, the specific NLRP3 pathways targeted, and the key findings.

**Table 5:** Summary of studies done so far on nanosized drug delivery systems targeting NLRP3 inflammasome for enhancing the treatment of sepsis.

Nanocarrier	Therapeutic agent	In vitro/In vivo models	NLRP3 pathway targeted	Type of treatment	Key findings from the studies	Refs.
Inorganic NPs (Iron NPs)	Iron	CLP‐sepsis mice model	Expression of mRNA in NLRP3, caspase‐1, and IL‐1β	Therapeutic model	NanoFe ○ Decreased levels of NLRP3 inflammasome markers. ○ Protected against myocardial injury in sepsis via attenuation of inflammation.	[120]
Inorganic NPs (Mesoporous silica NPs)	L‐arginine	CLP‐sepsis mice model	ROS scavenging NLRP3 and NF‐κB P65 expressions	Post‐exposure prophylaxis model	PCM‐MSN@LA ○ Significantly reduced inflammation and ROS production, improving myocardial function in the early phase of sepsis.	[121]
Inorganic NPs (Ca carbonate Nps)	Colchicine	CLP‐sepsis rat model	TLR4/NFκB/NLRP3 signaling pathway	Therapeutic model	ColCaNPs ○ Downregulated TLR4, p‐NF‐κB, and NLRP3 expressions. ○ Suppressed NLRP3‐mediated cardiac pyroptosis.	[122]
Protein NPs (Se@BSA NPs)	Selenium (Se)	HK‐2 cells IRI‐AKI mice model	GPx‐1/ NLRP3/ Caspase‐1 pathway	Therapeutic model	Se@BSA NPs ○ Decreased reactive oxygen species (ROS). ○ Increased glutathione peroxidase (GPx)‐1 levels. ○ Inhibited NLRP3 inflammasome activation.	[123]
Protein NPs	Doxycycline	Mouse ALI model	Inflammasome effectors pathway	Prophylactic model	Doxy NPs ○ Significantly reduced the levels of NLRP3, caspase‐1 and IL‐1β (p = 0.0029).	[124]
Dendrimer (Sr‐G4‐PEG)	Thioether moeities	Beas‐2b cells ALI mice model	ROS scavenging and cytokine sequestration	Therapeutic model	Sr‐G4‐PEG scavenged ROS and sequestered TNF‐𝛼, IL‐6, and IL‐1𝛽) hence; ○ Alleviated alveolar bleeding. ○ Reduced inflammatory cell infiltration. ○ Suppressed release of inflammatory cytokines.	[125]
Telodendrimer	Tetracycline‐3	LPS‐murine model	NLRP3 inflammasome / caspase‐1 pathway	Post‐exposure prophylaxis model	nCMT3 ○ Significantly attenuated markers of lung injury‐ TNF‐𝛼, IL‐1β, IL‐6, IL‐18 and reduced lung injury.	[126]
Lipid‐coated NPs	NAD(H) Rifampicin	BMDMs Endotoxemia mouse model Polymicrobial bacteremia model	ASC speck formation STAT‐1/IFN‐β pathway	Therapeutic model	NAD+‐LP‐CaP/ NADH‐LP‐MOF ○ Attenuated inflammation and pyroptosis. ○ Superior therapeutic efficacy in treating LPS‐induced endotoxemia, with a 100% survival rate. ○ Insignificant efficacy in treating polymicrobial bacteremia when NAD+‐LP‐CaP is used alone. ○ Superior efficacy in treating polymicrobial bacteremia when combined with rifampicin (NAD+‐Rif‐LP‐CaP), with a 90% survival rate.	[127]
Liposomes	Glibenclamide	THP‐1 cells	ASC/NLRP3/pro‐caspase 1. miR‐223‐3p expression	Post‐exposure prophylaxis model	GNVs ○ Significantly reduced ASC‐speck oligomerization, NLRP3 transcription and secretion of caspase 1 and IL‐1β.	[128]
Liposomes	*Fto*‐siRNA Entacapone	BMDMs Mice sepsis model	FoxO1/NF‐kB signaling	Prophylactic model	○ Silencing *Fto* suppressed production of IL‐1β that is NLRP3 inflammasome‐mediated. ○ Higher survival rate in mice with LPS induced septic shock.	[129]
Liposomes	MCC 950 Disulfiram	BMDMs	NLRP3 inflammasome / gasdermin D pathway	Therapeutic model	MCC–DSR Nps ○ Inhibited activation of NLRP3 to active form. ○ Significantly suppressed caspase‐1 and IL‐1β production. ○ Substantially inhibited activation of gasdermin D.	[130]
Polymer NPs	Dexamethasone (DEX)	ALI mouse model	ROS‐NLRP3 signaling pathway	Post‐exposure prophylaxis model	PFTU@DEX NPs ○ Strongly suppressed NLRP3, caspase and IL‐1β expression. ○ Eliminated excess ROS, thus inhibiting activation of NLRP3 inflammasome.	[131]
Polymer NPs	Azithromycin (AZI) Ibuprofen (IBF)	ALI mouse model	ROS‐NLRP3 signaling pathway	Therapeutic model	AZI+IBF@NPs ○ Eliminated excess ROS, inhibiting NLRP3 inflammasome. ○ Suppressed NLRP3 inflammasome dependent IL‐1β levels.	[132]
Hybrid NPs	Shikonin (Shik)	CLP mice sepsis model	AMPK/SIRT1 pathway	Prophylactic model	Zn‐Shik‐PEG NPs ○ Activated AMPK/SIRT1 signaling inhibiting NLRP3 inflammasome activation in vivo. ○ Exhibited ROS scavenging.	[133]
Dopamine‐based NPs	Dopamine SW033291	ALF mouse model	JNK signaling, caspase and NLRP3 pathway	Therapeutic model	SW@DSeSeD ○ Significantly suppressed p‐JNK, caspase‐3 and NLRP3 proteins expression ○ Inhibited IL‐1β which is NLRP3 inflammasome dependent.	[134]
DNA nanoprism	Buformin p65 siRNA	RAW 264.7 cells/ ALI mouse model	AMPK‐NF‐κB p65‐NLRP3 pathway	Therapeutic model	PMBuf‐p65 ○ Significant reduction in the inflammatory cytokines IL‐1β and IL‐18 ○ Substantial suppression of NLRP3‐mediated pyroptosis and amelioration of ALI	[135]

AMPK: Adenosine monophosphate‐activated protein kinase; BSA: Bovine serum albumin; BALF: Bronchoalveolar lavage fluid; ASC: Adapter protein apoptosis‐associated speck‐like protein containing a CARD; ALF: Acute liver failure; CLP: Cecal ligation puncture; ROS: Reactive oxygen species; NF‐κB: nuclear factor κB; p65: AKI: Acute kidney injury; LPS: Lipopolysaccharide; GPx‐1: Glutathione peroxidase 1; BMDMs: Bone‐marrow‐derived macrophages; ALI: Acute lung injury; JNK: c‐Jun amino‐terminal kinases; PEG: Polyethylene glycol; Fto: Fat mass and obesity‐related protein.

#### 3.3.1 Inorganic‐Based Nanocarriers

Inorganic‐based nanocarriers represent a novel approach to target and modulate the NLRP3 inflammasome, leveraging their unique physicochemical properties. To date, several inorganic nanocarriers, including iron nanoparticles, mesoporous silica nanoparticles, and calcium carbonate nanoparticles, have been developed and assessed for their potential to inhibit NLRP3 inflammasome activation and protect against septic myocardial injury. However, there remains significant potential to explore additional inorganic nanocarriers for targeting not only septic myocardial injury but also other organ injuries related to sepsis. In this subsection, we review recent studies on inorganic nanocarriers, categorized by the type of inorganic material used in their design.

**Figure 8:**
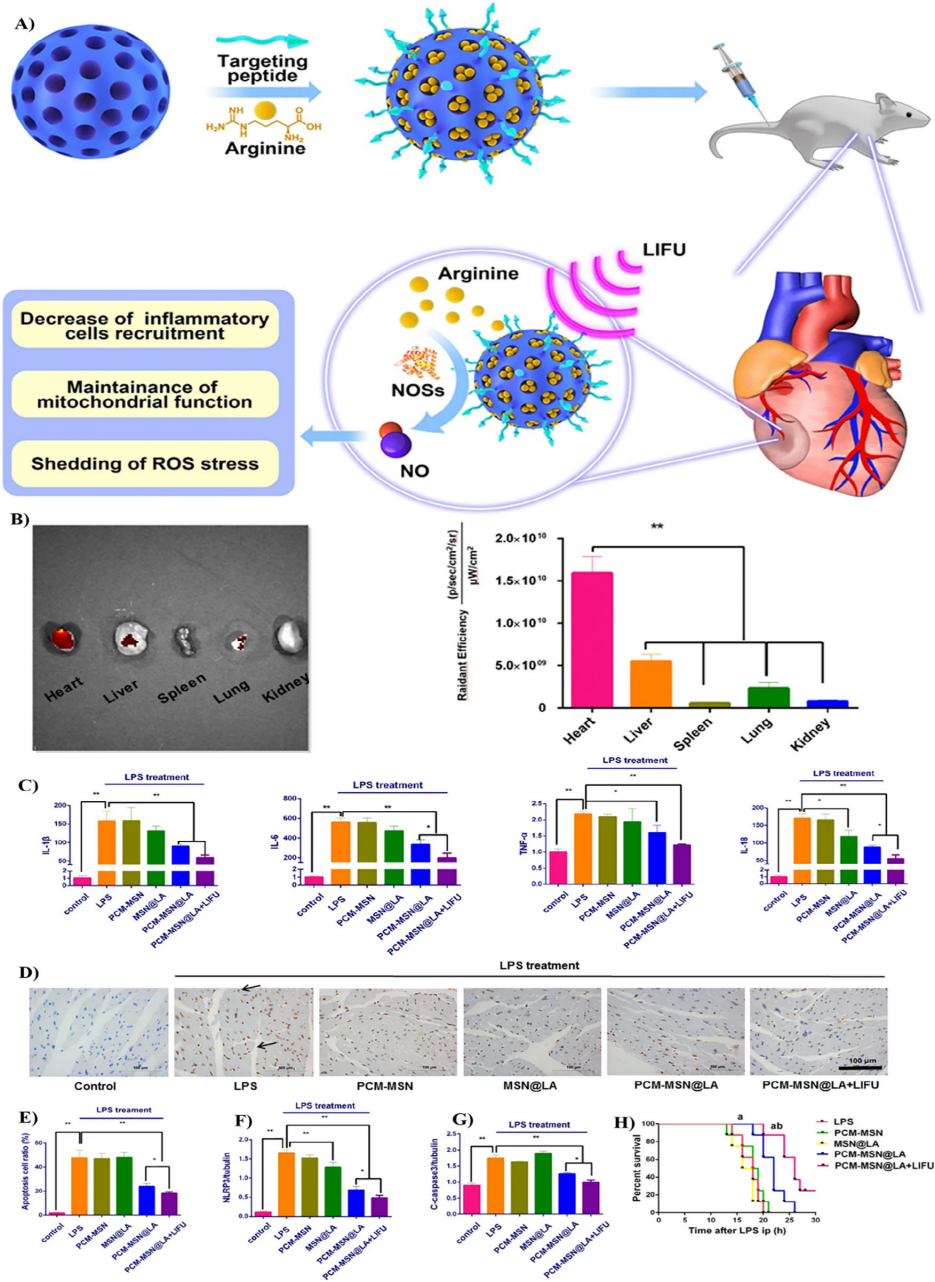
Heart‐targeted amelioration of sepsis‐induced myocardial dysfunction by microenvironment‐responsive nitric oxide nanogenerators in situ. A) Design of PCM‐carried and L‐arginine loaded porous MSN (PCM‐MSN@LA), combined with the LIFU, preventing myocardial dysfunction. B) In vivo cardiac targetability of PCM‐MSN@LA. C) Sequestration of major inflammatory cytokines by PCM‐MSN@LA following in vivo LPS stimulation. D) TUNEL staining of cardiac sections from mice subjected to different treatments. Quantitative analysis of E) Apoptotic cells, F) NLRP3 expression and G) Caspase 3 protein, following various treatments. H) Survival analysis of mice with severe sepsis following different treatment regimens. Adapted under the terms of the Creative Commons CC By 4.0 license.^[^ ^121^ ^]^ Copyright 2023, Ouyang et al.

Using iron, Wang et al.^[^ ^120^ ^]^ fabricated iron nanoparticles (nanoFe) and assessed their protective effect in CLP‐induced septic myocardial injury. The nanoFe exhibited favorable physicochemical properties and were found to be non‐cytotoxic. In vivo studies showed that the treatment of CLP mice with nanoFe significantly silenced the mRNA expression of inflammatory markers associated with NLRP3 inflammasome activation, thereby attenuating myocardial inflammation and preventing injury. This promising finding highlights the potential of iron‐based nanocarriers in modulating inflammation and providing cardioprotective effects during sepsis. Using another inorganic material, mesoporous silica, Ouyang et al.^[^ ^121^ ^]^ developed mesoporous silica nanoparticles (MSNs) to deliver L‐arginine (LA), a nitric oxide (NO) donor, to the impaired myocardial microenvironment in sepsis. The MSNs, as shown in **Figure** **8**A, were functionalized with a targeting peptide (PCM), forming the PCM‐MSN@LA complex, which was further guided to the site of myocardial injury using low‐intensity focused ultrasound (LIFU). The PCM‐MSN@LA+LIFU demonstrated favorable physicochemical properties (size: 186.67 nm, zeta potential: ‐6.23 mV), good biosafety, and controlled release of the therapeutic agent. In addition, the system showed significant potential in reducing ROS production, suppressing NLRP3 expression, decreasing cardiomyocyte apoptosis, and improving survival in the sepsis model (Figure 8), illustrating the promising therapeutic effects of MSNs in sepsis‐related myocardial injury.

By use of calcium carbonate, Wang et al.^[^ ^122^ ^]^ designed calcium carbonate nanoparticles loaded with colchicine (ColCaNPs) to offer protection against myocardial injury following myocardial infarction. These ColCaNPs exhibited appropriate safety profiles and suitable physicochemical properties (size: 243 nm, PDI: 0.11). The nanoparticles effectively inhibited NF‐κB and NLRP3 expression, leading to a reduction in the inflammatory response and myocardial injury, underscoring the potential of calcium carbonate nanoparticles in targeting NLRP3 inflammasome activation and protecting against cardiac damage. However, the study is limited by the lack of data supporting the release kinetics of colchicine, which is an important factor for evaluating the therapeutic effectiveness of the nanosystem.

Collectively, these studies highlight the potential of inorganic nanocarriers in targeting the NLRP3 inflammasome and offering cardioprotective effects in sepsis‐induced myocardial injury. While current research predominantly focuses on myocardial damage, expanding the application of these nanocarriers to target other organs affected by sepsis holds substantial promise. Future studies should aim to further optimize the design of these nanocarriers and explore their efficacy in treating multi‐organ dysfunction in sepsis. Additionally, investigating other inorganic nanocarriers, such as metal nanoparticles, and exploring their integration with polymeric and lipidic systems to form hybrid nanocarriers represents an exciting avenue for future research. These hybrid systems could enhance the inhibition of NLRP3 inflammasome activation and offer more effective therapeutic strategies for sepsis and its associated complications.

#### 3.3.2 Protein‐Based Nanocarriers

Protein‐based nanocarriers, through tailored design and functionalization, offer an effective strategy for targeting and modulating the NLRP3 inflammasome. One example is the use of bovine serum albumin (BSA), which has been employed in various studies to enhance the therapeutic efficacy of nanoparticles. For instance, targeting GPx‐1/NLRP3/Caspase‐1 pathway, Wang et al.^[^ ^123^ ^]^ fabricated selenium‐bovine serum albumin nanoparticles (Se@BSA NPs) and assessed their mitigative effects on AKI. Glutathione peroxidase 1 (GPx‐1), which has renoprotective properties, plays a key role in suppressing NLRP3 inflammasome activation. By increasing GPx‐1 levels through selenium supplementation, the Se@BSA NPs helped reduce the activation of pro‐inflammatory mediators and prevent the progression of ischemic injury. The Se@BSA NPs were noncytotoxic and nonhemolytic at doses up to 200 µg/mL and mainly accumulated in the liver and kidney tubules, as demonstrated by fluorescein isothiocyanate (FITC) labeling results. In an AKI mouse model, treatment with Se@BSA NPs led to a significant reduction in NLRP3 expression compared to untreated controls (P < 0.001). In vitro experiments using HK‐2 cells exposed to hypoxia/reoxygenation, mimicking IRI, were consistent with these findings. Moreover, treatment with Se@BSA NPs also resulted in a notable decrease in ROS levels, from 13,662.4 ± 1046.9 to 8303.9 ± 406.7, suggesting that the nanoparticles modulated oxidative stress. Furthermore, Se@BSA NPs significantly increased GPx‐1 levels both in vitro and in vivo. Together, these results demonstrate that Se@BSA NPs can modulate GPx‐1 levels, thereby suppressing NLRP3 inflammasome activation and providing a therapeutic effect in IRI‐induced AKI.

In another study, Arora and Vyavahare^[^ ^124^ ^]^ also fabricated doxycycline‐loaded BSA nanoparticles functionalized with elastin antibodies to target acute ALI. This nanosystem showed potential in downregulating the levels of inflammasome components such as NLRP3, caspase‐1, and IL‐1β, suggesting its ability to modulate inflammasome activation in sepsis‐induced lung injury. However, we believe that the characterization of the nanosystem in this study was insufficient to fully assess its potential. For instance, important data on the physicochemical properties, in vivo biodistribution, and biosafety of the system were lacking. Specifically, cytotoxicity and hemolysis assays, which are crucial for evaluating safety and biocompatibility, were not provided. Moreover, the lung injury in the mouse model was induced using a small dose of elastase, which may not have caused sufficient pathological changes to assess the full therapeutic potential of the nanocarrier. Additionally, the study employed a high dose of doxycycline (equivalent to an adult dose of 600 mg), which raises concerns about potential toxicity, which has not been evaluated. Furthermore, although the nanoparticles were functionalized with elastin antibodies, no studies were performed to assess their biodistribution or their specific binding to lung tissue. Therefore, while the study provides some preliminary insights into the potential of this nanosystem, we contend that the findings are not conclusive. Further studies are needed to confirm the accumulation of the nanocarrier at the target site, elucidate its biosafety in both in vitro and in vivo models, and explore its pharmacokinetics and pharmacodynamics.

#### 3.3.3 Dendrimer‐Based Nanocarriers

Dendrimers, as hyperbranched macromolecules, offer exceptional multifunctionality for carrying a variety of therapeutic agents. Recent studies have highlighted their potential in targeting and modulating inflammasomes, particularly in the context of ALI. In this regard, Jiang et al.^[^ ^125^ ^]^ developed a thioether‐enriched dendrimer, Sr‐G4‐PEG, designed to scavenge ROS and proinflammatory cytokines in ALI. The Sr‐G4‐PEG exhibited excellent biocompatibility and preferential accumulation in lung epithelial cells, particularly in areas of injury. In an in vivo mice model of LPS‐induced ALI, Sr‐G4‐PEG significantly inhibited pyroptosis markers (NLRP3, caspase‐1, IL‐1β, and GSDMD‐N), suggesting its potential to reduce inflammasome activation. Additionally, Sr‐G4‐PEG demonstrated excellent ROS scavenging as indicated by less fluorescence intensity in the lungs of mice treated with Sr‐G4‐PEG. The incorporation of thioether groups in the dendrimer structure played a crucial role in enhancing responsiveness to oxidative stress, enabling targeted and controlled drug delivery through selective cleavage under inflammatory conditions. This innovative strategy offers significant promise for improving therapeutic outcomes in a range of inflammatory diseases associated with oxidative stress and inflammasome activation.

In a similar study, Meng et al.^[^ ^126^ ^]^ developed a telodendrimer loaded with modified tetracycline (nCMT‐3) to improve its bioavailability in the lungs for the treatment of ALI. The telodendrimer demonstrated favorable physicochemical properties, including suitable size and stability, and was shown to be biocompatible. Following intratracheal administration, nCMT‐3 concentration was sustained in the lungs and kidneys for over 8 h. Western blot analysis revealed significant reductions in levels of caspase‐1 and NLRP3, two key components of the inflammasome. Moreover, analysis of BALF indicated lower levels of IL‐1β and IL‐18, further supporting the inhibitory effects of the system on the NLRP3 inflammasome/caspase‐1 pathway. These findings suggest that the telodendrimer‐based delivery system effectively targets the NLRP3 inflammasome and could offer a promising therapeutic strategy for treating ALI and other sepsis‐related organ injuries.

Overall, dendrimer‐based nanocarriers, with their versatile surface functionalization and enhanced bioavailability, hold significant promise for improving drug targeting and inhibiting NLRP3 inflammasome activation. These nanocarriers represent a promising approach for developing more effective treatments for inflammatory conditions linked to inflammasome dysregulation, such as ALI and other acute inflammatory diseases.

#### 3.3.4 Lipid‐Based Nanocarriers

Lipid‐based nanocarriers are emerging as powerful vehicles for enhancing the targeted delivery of therapeutics aimed at modulating the NLRP3 inflammasome, offering promise for both monotherapy and dual therapy approaches. This section reviews recent studies employing lipid‐based nanocarriers in the context of inflammasome inhibition against sepsis.

For delivery of a single agent, Ye et al.^[^ ^127^ ^]^ loaded an immunomodulator, NAD^+^(H), into a lipid nanocarrier for intracellular delivery aimed at replenishing NAD^+^(H) levels that are depleted during inflammation and oxidative stress, particularly in the context of sepsis. The lipid nanoparticles demonstrated good biocompatibility with a loading efficiency exceeding 60%, and pH‐responsive release behavior. The therapeutic efficacy of the NAD(H)‐loaded nanocarriers was assessed using LPS‐stimulated BMMΦs, where pyroptosis, caspase‐1 activation, and IL‐1β production were measured as indicators of NLRP3 inflammasome activation. The results revealed a significant reduction in caspase‐1 and IL‐1β levels compared to controls (p < 0.0001), indicating effective inhibition of NLRP3 inflammasome. Additionally, NF‐κB p65 translocation, a key mediator of inflammation, was significantly reduced in the NAD(H)‐treated group. The NAD(H)‐loaded NPs alone were effective against LPS‐induced endotoxemia, with 100% mouse survival, but not against the CLP‐induced sepsis/ polymicrobial bacteremia model. However, when combined with rifampicin, there was 90% survival in the polymicrobial bacteremia model. The improved outcome observed with the NAD(H)‐Rif‐loaded NPs likely results from both enhanced energy metabolism (via NAD(H)) and increased antibiotic bioavailability in infected tissues (via nanoparticle‐mediated delivery and improved tissue permeability).

In a similar approach, Mancuso et al.^[^ ^128^ ^]^ utilized bi‐functionalized liposomes to encapsulate glibenclamide, characterized by limited solubility, to enhance its bioavailability for targeting neuroinflammation. The nanosystem was assessed for its ability to modulate the NLRP3 inflammasome via the reduction of NLRP3‐related cytokines and gene expression in the THP‐1 cell model. The glibenclamide‐loaded liposomes significantly downregulated miR‐223‐3p and reduced the secretion of inflammasome effectors, such as caspase‐1 and IL‐1β. However, a critical limitation of this study was the lack of essential characterization data, such as physicochemical properties, cytotoxicity, and hemolysis assays, raising concerns about the system's biocompatibility and safety profile. These gaps in the data hinder the full assessment of the nanosystem's potential for clinical application.

Also, Luo et al.^[^ ^129^ ^]^ loaded *Fto*‐siRNA and entacapone separately into liposomes for the treatment of septic shock through suppression of NLRP3‐associated inflammation. The liposomes were shown to be non‐cytotoxic, and their therapeutic effect was evaluated in an endotoxemic mouse model of sepsis. Western blot analysis revealed that Fto‐siRNA liposome treatment significantly suppressed the expression of the p65 gene, potentially inhibiting the FoxO1/NF‐κB signaling pathway and thereby reducing NLRP3 inflammasome‐mediated IL‐1β production. Similarly, entacapone‐loaded liposomes also led to reduced inflammasome activity and cytokine release. While these results suggest a promising therapeutic strategy, the study lacked detailed information on the liposomes' physicochemical properties, which critically influence liposomal stability, biodistribution, and cellular uptake, was not documented, which are critical for understanding their pharmacokinetics and efficacy in vivo.

By employing a dual therapy strategy, Nandi et al.^[^ ^130^ ^]^ designed a liposomal nanocarrier loaded with MCC 950, a selective NLRP3 inhibitor, and disulfiram, a GSDMD activation inhibitor, for the treatment of septic peritonitis. The nanosystem was well characterized, with a suitable size, zeta potential, and cell viability greater than 75%. In vivo studies demonstrated that the dual therapy approach significantly reduced levels of caspase‐1 and IL‐1β. Moreover, the liposomal nanocarrier improved survival rates in mice exposed to high doses of LPS (50 mg/kg), reduced inflammasome markers and facilitated complete recovery from septic shock even at lower doses. However, despite disulfiram being a GSDMD inhibitor, the western blot data showed no significant inhibition of GSDMD by either free disulfiram or disulfiram NPs. This may be attributed to the timing of treatment or other experimental factors that should have been pointed out by the authors.

Collectively, these studies highlight the potential of lipid‐based nanocarriers in enhancing drug delivery and improving therapeutic outcomes for inflammasome‐mediated conditions. Ongoing research into optimizing these systems will likely lead to improved treatment modalities for inflammatory diseases associated with NLRP3 activation, including sepsis.

#### 3.3.5 Polymer‐Based Nanocarriers

Polymer‐based nanocarriers that respond to oxidative stress by scavenging ROS and inhibiting the NLRP3 inflammasome have been widely developed for treating conditions characterized by oxidative stress and inflammation, such as sepsis‐related ALI.

For instance, Muhammad et al.^[^ ^131^ ^]^ designed a ROS‐responsive polymeric nanocarrier (PFTU) loaded with dexamethasone (PFTU@DEX NPs) for the treatment of ALI. The PFTU@DEX NPs exhibited favorable physicochemical properties, including non‐cytotoxicity and non‐hemolytic activity, and demonstrated accelerated release of dexamethasone in a ROS‐rich environment. The potential of this nanosystem was evaluated using both in vitro models (RAW 264.7 and A549 cells) and an in vivo ALI model in mice. Inflammatory markers, including NLRP3, caspase‐1, and IL‐1β, were measured after LPS stimulation and treatment with PFTU@DEX NPs or controls. LPS stimulation led to an upregulation of NLRP3, caspase‐1, and IL‐1β levels in lung tissue. However, treatment with PFTU@DEX NPs significantly suppressed these markers, effectively reducing inflammation by promoting macrophage polarization from the pro‐inflammatory M1 phenotype to the anti‐inflammatory M2 phenotype. In another study, Muhammad et al.^[^ ^132^ ^]^ employed the same ROS‐responsive polymeric nanocarrier (PFTU) to co‐load azithromycin and ibuprofen (AZI+IBF@NPs) for the simultaneous eradication of bacteria and reduction of inflammation in ALI. The AZI+IBF@NPs displayed favorable physicochemical properties (size: 292 nm, zeta potential: −19 ± 0.4 mV), were non‐cytotoxic, and exhibited a hemolysis rate of <5%. Like the previous formulation, this nanosystem showed significant potential for inhibiting NLRP3‐related inflammation when tested using in vivo mouse models. Despite the promising results, the drug release studies from these nanocarriers indicated that the release was not sustained. Thus, we believe that the nanosystems could further be improved to achieve sustained release, which will be beneficial in the reduction of dosing frequency.

Guo et al.^[^ ^133^ ^]^ developed a hybrid metal‐polyphenol nanocarrier composed of zinc and shikonin (Zn‐Shik‐PEG NPs) to scavenge ROS and inhibit the NLRP3 inflammasome against sepsis. The Zn‐Shik‐PEG NPs were well characterized, with a size of 30 nm and cell viability of >75%. To investigate whether the Zn‐Shik‐PEG NPs inhibit the NLRP3 inflammasome through the AMPK/SIRT1 pathway, RAW 264.7 cells were stimulated with LPS, and the expression of AMPK, p‐AMPK, SIRT1, and NLRP3 was quantified. Western blot analysis revealed significant suppression of p‐AMPK and SIRT1, along with an increase in NLRP3 expression. Treatment with Zn‐Shik‐PEG NPs partially reversed these changes. However, despite the promising findings, the use of Raw264.7 cells is not suitable for studying NLRP3 inflammasome activation. The observed reduction in pro‐inflammatory mediators likely results from an NLRP3 inflammasome‐independent pathway.^[^ ^136^ ^]^ Therefore, further studies are needed to validate these results using more appropriate in vitro cell models, such as THP‐1 cells. The therapeutic efficacy of the nanosystem was further assessed in a sepsis model using LPS/CLP‐induced mice, where treatment with Zn‐Shik‐PEG NPs resulted in improved inflammation scores compared to controls. However, several concerns remain. Cell viability assays showed that bare shikonin is cytotoxic in a concentration‐dependent manner, with concentrations as low as 10 µg/mL resulting in severe toxicity (cell viability < 10%). Additionally, hemolysis data were not provided. These findings suggest that the biosafety and biocompatibility of the Zn‐Shik‐PEG NPs may be compromised under conditions of premature release or off‐target distribution, necessitating further investigation into their safety profile.

In summary, these studies highlight the potential of polymer‐based nanocarriers in targeting inflammation through ROS scavenging and NLRP3 inflammasome inhibition in sepsis and related injuries. These nanocarriers offer the advantage of selectively targeting inflamed tissues, improving drug delivery, and helping to restore homeostasis in oxidative stress conditions. However, further research is needed to optimize these systems, particularly with regard to sustained drug release, safety, and efficacy, to develop more effective treatments for NLRP3 inflammasome‐associated disorders.

#### 3.3.6 Other Nanocarriers

Recent studies have also explored other nanocarriers, such as dopamine‐based systems and DNA nanoprism‐based carriers, for their potential in targeting and inhibiting the NLRP3 inflammasome. These nanocarriers have been utilized for dual therapy in a variety of contexts, as described below.

To co‐deliver dopamine and SW033291, an NLRP3 inflammasome inhibitor, Zhan et al.^[^ ^134^ ^]^ designed a dopamine‐based nanodrug (SW@DSeSeD) for inhibiting NLRP3 inflammasome, for the treatment of acute liver injury. The SW@DSeSeD nanoparticles were responsive to reactive oxygen species (ROS), displayed favorable physicochemical properties, and exhibited non‐cytotoxicity. To assess the anti‐inflammatory potential of the nanocarrier, the nanoparticles were incubated with LPS‐stimulated RAW 264.7 cells for 20 h. IL‐1β and TNF‐α levels were then quantified using ELISA. Additionally, the levels of caspase‐3, p‐JNK, and NLRP3 in liver tissues from mice with LPS‐induced acute liver failure were assessed. Both in vitro and in vivo results showed a significant reduction in the levels of pro‐inflammatory mediators, indicating that the SW@DSeSeD nanoparticles effectively attenuated inflammation.

**Figure 9:**
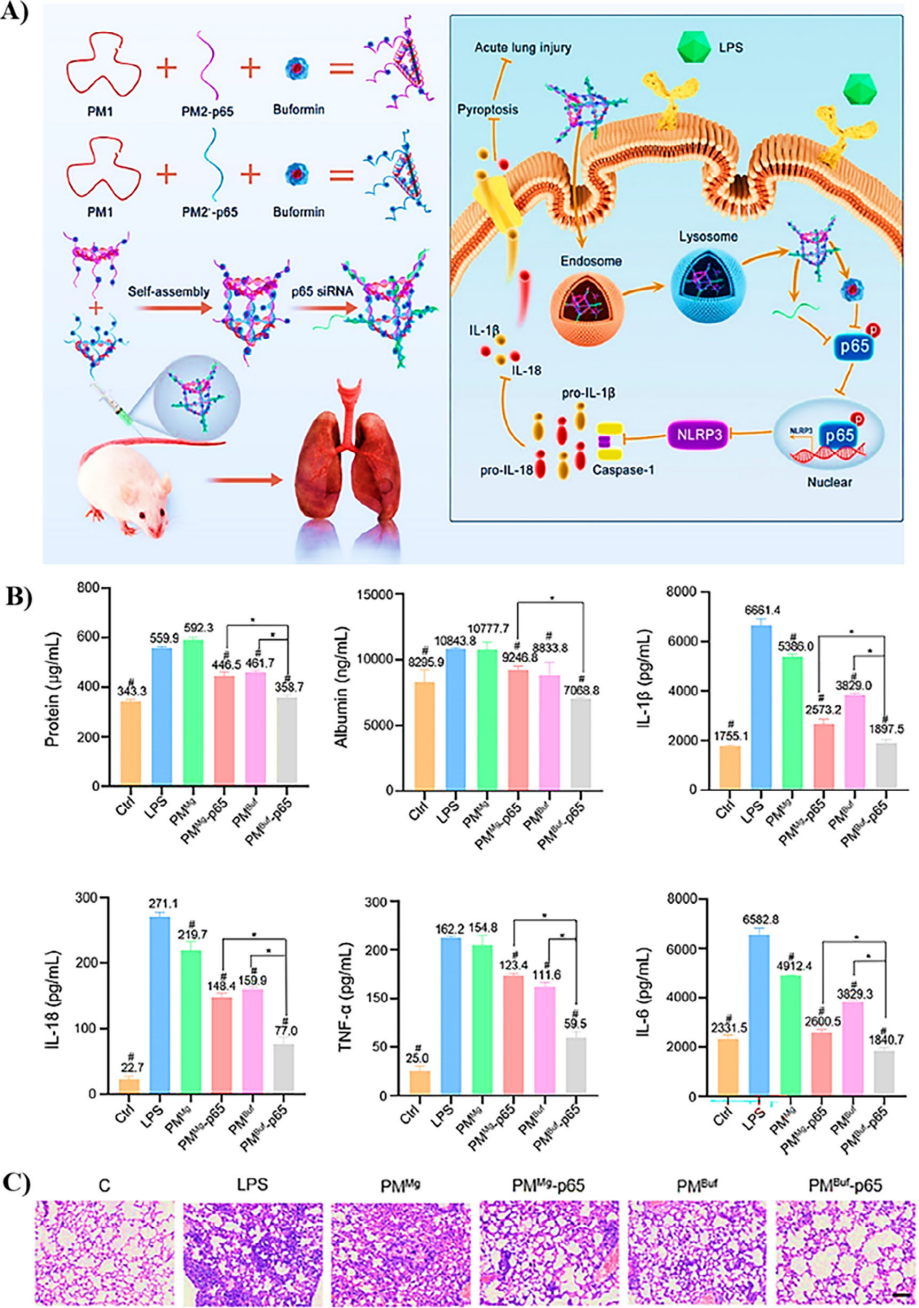
p65 siRNA‐loaded buformin/DNA nanoprisms alleviate acute lung injury through inhibiting NLRP3‐mediated pyroptosis. A) Design and mechanism of action of PMmg‐p65 in anti‐pyroptosis relative to NLRP3 activation. B) Quantification of proteins and cytokines in an in vivo model of ALI following treatment with PMmg‐p65, as measured by ELISA. C) Histological observations of lung sections following H&E staining post‐treatment. Adapted with permission.^[^ ^135^ ^]^ Copyright 2023, American Chemical Society.

Similarly, to co‐deliver buformin and six p65 siRNA, You et al.^[^ ^135^ ^]^ designed a DNA nanoprism as a nanocarrier (PM^Buf^‐p65) to inhibit NLRP3‐mediated pyroptosis in ALI. The nanosystem demonstrated adequate stability, biocompatibility and high cellular uptake. In vitro and in vivo assessments were conducted using LPS + ATP‐treated RAW 264.7 cells and ALI mouse models, respectively. ALI mouse model showed synergistic effects between buformin (an AMPK pathway activator) and p65 siRNA (which silences p65 mRNA expression), leading to a significant reduction in the inflammatory cytokines IL‐1β and IL‐18. This resulted in substantial suppression of NLRP3‐mediated pyroptosis and amelioration of ALI. **Figure** **9** illustrates the design and the inflammation inhibitory effects of PM^Buf^‐p65 in the ALI mouse model.

The exploration of alternative nanocarriers, such as dopamine‐based systems and DNA nanoprism carriers, represents a promising frontier in the targeted inhibition of the NLRP3 inflammasome. These findings inspire further research into novel drug delivery systems, such as exosomes, which could offer additional therapeutic benefits.

In summary, the dual nature of the NLRP3 inflammasome, acting as both a defender against infections and a contributor to chronic inflammation, requires careful modulation for optimal therapeutic outcomes. The therapeutic timing of NLRP3 inflammasome modulation is critical. Targeted NLRP3 inhibition during the early to mid‐phases of sepsis progression may help restore immune equilibrium by reducing pro‐inflammatory cytokine release while avoiding suppression of anti‐inflammatory mediators. Nanocarriers that can modulate the NLRP3 inflammasome pathway, suppress its activation, and mitigate downstream effects can significantly reduce the expression of key inflammatory markers such as caspase‐1 and IL‐1β. This, in turn, can help attenuate inflammatory responses. Collectively, these studies underscore the diverse approaches and potential of nanocarriers in targeting NLRP3 inflammasome‐driven inflammation, particularly in conditions such as sepsis, and highlight promising therapeutic avenues for managing a variety of inflammatory disorders, like sepsis.

## 4 Conclusion, Limitations, and Future Perspectives

Sepsis is a critical, life‐threatening condition characterized by systemic inflammation that profoundly disrupts both innate and adaptive immunity. Despite significant advances in our understanding of sepsis pathophysiology, current treatment strategies have yet to substantially improve patient outcomes, and the high mortality rate (38.6% to 80%) remains a major concern. Recent developments in nanocarrier‐based drug delivery systems offer a promising shift in the treatment paradigm, with the potential to address both the underlying inflammation and immune dysregulation seen in sepsis. Targeting PRRs for therapeutic modulation, including TLRs, and NLRs, could be key to restoring immune balance and reducing sepsis‐related complications.

Although still in its early stages, research on engineered nanocarriers targeting PRRs, such as TLRs and NLRs, particularly the NLRP3 inflammasome, has advanced considerably. Several types of nanocarriers, including inorganic, polymeric, lipid‐based, protein‐based, dendrimer, and nanoprisms, have been extensively studied for their ability to target these receptors and modulate the immune response in sepsis. Some of such nanocarriers utilize materials with inherent inhibitory activity against TLRs and NLRP3, while others are designed to deliver PRR inhibitors in combination with antibiotics or anti‐inflammatory agents. These systems have shown considerable promise in reducing inflammatory markers (e.g., TNF‐α, IL‐6, IL‐1β, IL‐10, IL‐18, pro‐caspase‐1, and caspase‐1) and improving clinical outcomes in experimental sepsis models.

A closer analysis of the literature reveals that most studies focus on conventional biomimetic nanocarriers designed to target TLR‐ and NLRP3‐mediated inflammatory pathways. However, there is a notable lack of nanocarrier designs aimed at targeting other PRRs involved in recognizing a broader range of PAMPs, such as other NLRs (e.g., NOD1, NOD2, NLRC4, AIM2), as well as RLRs, CLRs, RAGE, and DNA‐sensing molecules, specifically for sepsis therapy. This gap presents an exciting opportunity for future research. Additionally, there is limited exploration of more advanced strategies combining stimuli‐responsive and biomimetic approaches to enhance targeted drug delivery against sepsis. These dual‐function nanocarriers, responsive to factors such as pH, ROS, redox conditions, or enzymatic activity, could offer more precise and selective therapeutic outcomes by enabling on‐demand drug release and modulation of immune responses. Thus, future research should prioritize the development of stimuli‐responsive and biomimetic nanocarriers that also target PRRs for sepsis therapy.

Additionally, while most current nanocarriers (more than 90% of reported studies) are designed to target PRRs involved in the recognition of Gram‐negative bacterial toxins, such as TLR4, there is a notable lack of focus on PRRs responsible for recognizing Gram‐positive bacteria, such as TLR2. This gap presents an exciting opportunity for future research to explore novel TLR2 ligands and integrate them into nanocarriers for the treatment of sepsis caused by Gram‐positive bacteria, including MRSA. Furthermore, leveraging natural negative feedback mechanisms of PRR‐mediated inflammation through the design of extracellular decoys, TLR‐depleting molecules, or inflammasome inhibitors could open new avenues for developing innovative nanocarriers, thereby broadening therapeutic options.

An important and underexplored area in targeting PRRs in sepsis immunotherapy is the use of exosomes, small vesicles that carry a variety of bioactive molecules, including proteins, nucleic acids, and small RNAs. Exosomes play a crucial role in immune regulation and have been shown to interact with TLRs to elicit anti‐inflammatory responses. Their potential as drug delivery vehicles, capable of carrying antibiotics and anti‐inflammatory agents, could provide a novel, multi‐faceted approach to sepsis therapy.

Despite recent progress, several limitations persist in the design and application of nanocarriers targeting PPRs for sepsis treatment. A deeper understanding of the molecular behavior of these nanosystems in biological environments is necessary to optimize their design. The integration of artificial intelligence (AI) tools,^[^ ^137^ ^]^ including computational modeling, could accelerate the development of more targeted PRR ligands and improve nanocarrier design. Moreover, further studies on the pharmacokinetics, biosafety, and long‐term stability of these nanocarriers are essential to ensure their safe progression into clinical trials.

In our view, the successful translation of promising nanocarrier systems into clinical trials will require a coordinated global effort involving collaboration across public and private sectors, including academic institutions, regulatory bodies, and pharmaceutical companies. Several key challenges must be addressed, such as standardizing manufacturing processes, establishing rigorous quality control measures, developing predictive preclinical models, and overcoming regulatory barriers. These obstacles can be mitigated through strategies such as implementing industry‐wide guidelines and automation to enhance reproducibility and scalability, utilizing advanced analytics and real‐time quality control to ensure product consistency and safety, and employing humanized animal models and organ‐on‐chip technologies to improve the predictive accuracy of preclinical studies. Early engagement with regulatory agencies is also essential to facilitate more efficient approval pathways. Furthermore, fostering public‐private partnerships and securing targeted funding will be critical to support the translation of these technologies. Addressing these challenges through coordinated strategies will be pivotal for advancing nanocarrier‐based PRR‐targeted therapies into clinical practice, potentially offering novel treatment options for sepsis and other inflammatory diseases.

In conclusion, while the field of nanocarriers targeting PRRs for enhancing the treatment of sepsis is still in its early stages, it holds significant promise for modulating the inflammatory response and improving clinical outcomes. With continued advancements in nanotechnology, AI‐driven drug design, and high‐throughput screening methods, future research is poised to yield novel, intelligent nanocarriers with the potential to transform sepsis therapy. These innovations could not only provide more effective treatments for sepsis but also contribute to the broader goal of addressing a range of PRR‐related inflammatory diseases.

## Conflict of Interest

The authors declare no conflict of interest.
